# Mapping intersectional inequalities in biomarkers of healthy ageing and chronic disease in older English adults

**DOI:** 10.1038/s41598-020-69934-8

**Published:** 2020-08-11

**Authors:** Daniel Holman, Sarah Salway, Andrew Bell

**Affiliations:** 1 Department of Sociological Studies, University of Sheffield, Sheffield, UK; 2 Sheffield Methods Institute, University of Sheffield, Sheffield, UK

**Keywords:** Biomarkers, Diseases, Risk factors

## Abstract

Chronic diseases and their inequalities amongst older adults are a significant public health challenge. Prevention and treatment of chronic diseases will benefit from insight into which population groups show greatest risk. Biomarkers are indicators of the biological mechanisms underlying health and disease. We analysed disparities in a common set of biomarkers at the population level using English national data (n = 16,437). Blood-based biomarkers were HbA1c, total cholesterol and C-reactive protein. Non-blood biomarkers were systolic blood pressure, resting heart rate and body mass index. We employed an intersectionality perspective which is concerned with how socioeconomic, gender and ethnic disparities combine to lead to varied health outcomes. We find granular intersectional disparities, which vary by biomarker, with total cholesterol and HbA1c showing the greatest intersectional variation. These disparities were additive rather than multiplicative. Each intersectional subgroup has its own profile of biomarkers. Whilst the majority of variation in biomarkers is at the individual rather than intersectional level (i.e. intersections exhibit high heterogeneity), the average differences are potentially associated with important clinical outcomes. An intersectional perspective helps to shed light on how socio-demographic factors combine to result in differential risk for disease or potential for healthy ageing.

## Introduction

The huge and growing burden of chronic diseases and their inequalities amongst older adults is a significant public health challenge. Prevention and treatment of chronic diseases, and therefore attainment of the policy goal of healthy ageing, will benefit from insight into which population groups show greatest risk. Biological markers—objective measures of biological processes underlying healthy ageing and disease^1^—are key risk factors. Many of the current gaps in knowledge on healthy ageing and chronic disease inequalities centre around the complex interaction of sociocultural, political and biological factors^2^. However these inequalities have often been investigated according to single or at most a limited number of categories of difference at a time, such as gender, ethnicity or socioeconomic position (SEP). Such unidimensional approaches can inadvertently reinforce the intractability of inequalities and fail to provide evidence on how to intervene^3^. By focussing on single social attributes, there is a risk that the disadvantages faced by particular subgroups can be rendered invisible^4^.

Intersectionality has increasingly been seen as a promising way to advance health inequalities research and policy^5–8^. Its essence is that multiple social attributes overlap and interact with each other to drive health outcomes^3,9–11^. Different intersections defined by combinations of social attributes such as gender, ethnicity and SEP, are potentially associated with different (though overlapping) health outcomes and are subject to different causal processes driving these outcomes. An intersectional approach may help to advance understanding of the social processes and circumstances that create poor (or good) health as well as inform the design of policies and interventions for varied social contexts and for different population subgroups^12^.

A first step in intersectional health inequalities research is to socio-demographically ‘map out’ disparities according to multiple social attributes. So far, researchers have done this for various health outcomes, including depression^13^, body mass index^14^ and chronic obstructive pulmonary disease^15^. Here we extend this work by focussing on a key set of biomarkers of healthy ageing, elevated levels of which are associated with a range of chronic diseases and their longer term implications^16^. Intersectional mapping of biomarker outcomes illustrates how population health is mutually constituted by the social and biological^17^. Intersectional disparities in biomarkers would suggest that we can modify the social factors associated with intersectional positions/identities to reduce these disparities in order to reduce inequalities in healthy ageing. Currently, there is a lack of knowledge on the social distribution of biomarkers, as highlighted by the European Roadmap for Ageing Research^2^. By bringing an intersectionality lens to the analysis of biomarker data, we offer a novel approach to filling this gap in knowledge.

There are many unanswered questions regarding the way in which health, including its causes and consequences, is patterned intersectionally. Intersectional patterning may be present not only in the distribution of disease, but in risk factors and their social determinants, life course processes and dynamics, the lived experience of health and illness, healthy and functional ageing outcomes, and the social processes linking body processes to diagnosis. Researchers have only recent begun answering such questions, yet they might provide valuable knowledge for population health strategies^6^. There are a number of possibilities for intersectional patterning. For example, it might be that the disadvantage associated with certain social attributes such as ethnic minority status is offset by advantages in another attribute such as SEP. This offsetting might occur for men but not women. Such subgroup differences would suggest a need to understand the social forces driving outcomes for particular subgroups, and that remedial policies or interventions may be inefficient or even increase inequalities if they are based on a single axis of inequality.

An underlying dimension to intersectional patterning is whether dis/advantages are additive i.e. layer on top of each other, or have multiplicative effects i.e. have amplifying or attenuating effects, so that intersectional outcomes are different than expected given the referent attributes that comprise them^18,19^. For example, in the former, the effect of ethnic minority status on health is the same regardless of, say, SEP, and in the latter, the effect of ethnic minority status on health might be particularly pronounced for those with low SEP. Without testing for interaction effects, subgroup differences are assumed to be the result of additive effects only, which can lead to erroneous interpretations^20^. The necessity of testing for multiplicative effects to avoid such misinterpretation has often been taken to mean that intersectionality is falsified when only additive effects are present, when in fact, intersectionality is mainly a framework to understand heterogeneity and the social structures driving it^18^.

In this paper, we examine patterns of key biomarkers of healthy ageing and chronic disease across intersectional subgroups and seek to answer two research questions: 1. What are the extent/nature of intersectional differences in later life (50 +) biomarker measures? 2. Are these intersectional differences multiplicative or additive? We use data from two national surveys containing comparable biomarker data. We focus on a set of six biomarkers which were available in both surveys and are commonly accepted as biomarkers of healthy and functional ageing and predictors of chronic disease morbidity and mortality^16^. Social disparities in the selected biomarkers are often analysed in order to unpack the social to biological processes leading to health inequalities^21^. We analyse data from older adults aged 50 or over as this when many chronic diseases emerge.

## Methods

### Study design

**Figure 1:**
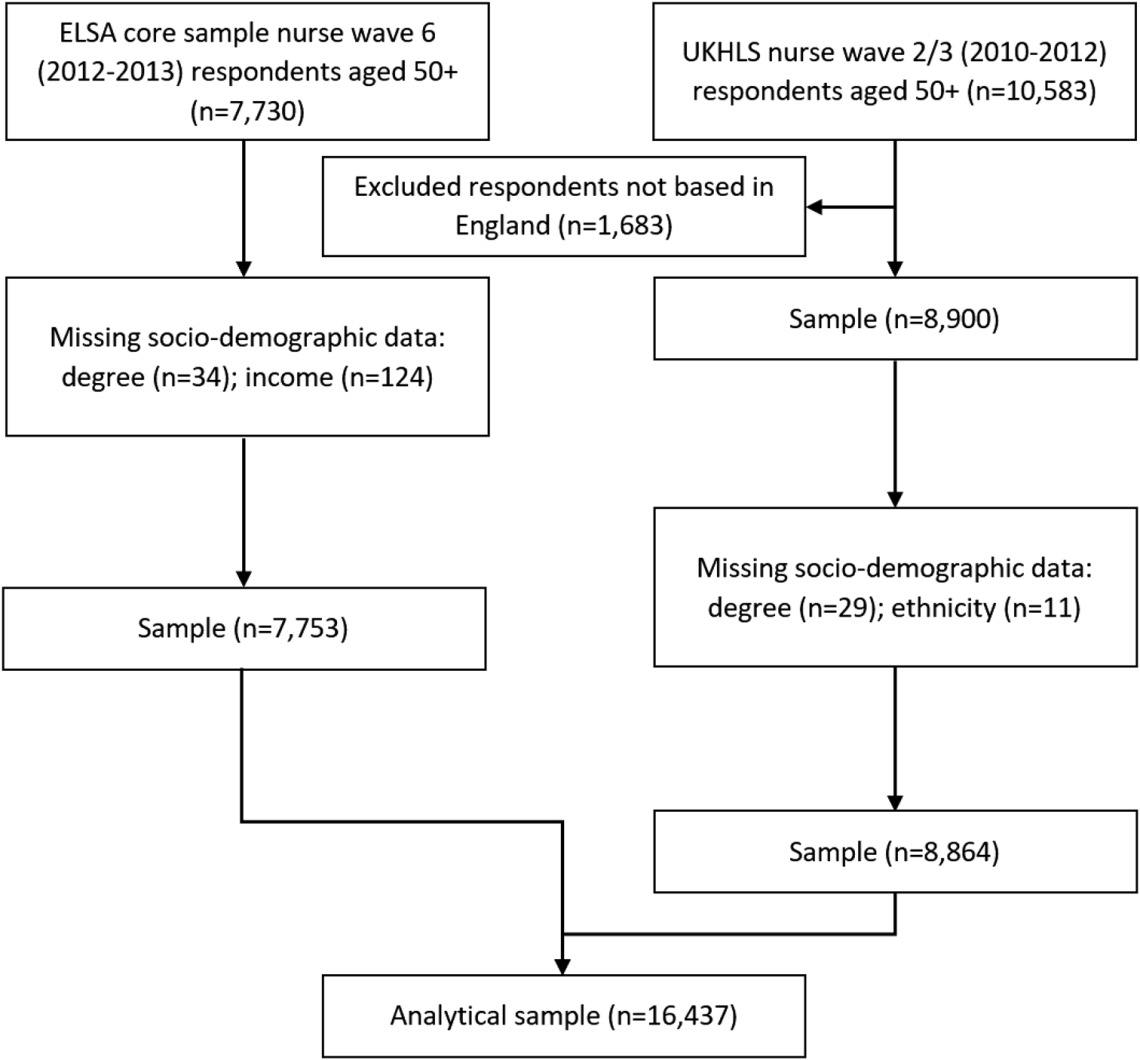
Flow chart diagram.

The English Longitudinal Study of Ageing (ELSA) is a biennial panel study of adults aged 50 + years living in England and includes biomarker collection every other wave^22^. Data from wave 6 (2012–2013) are analysed, including from the harmonised dataset^23^. Understanding Society: the United Kingdom Household Longitudinal Study (UKHLS) is a yearly household panel survey of people aged 16 + living in the United Kingdom. Data from the nurse health visit which took place across waves 2 and 3 in 2010–2012 are analysed. Data from the two surveys were pooled as shown in Fig. 1. Although ELSA and UKHLS have some differences in sampling design according to stratification and clustering, both surveys aimed for a nationally representative sample. As Table 1 and Supplementary Fig. 1 show, both surveys were highly similar in their socio-demographic composition and distribution according to the biomarkers analysed, though some small differences exist for ethnicity which we discuss in the limitations. After selecting respondents only resident in England in UKHLS, and excluding those aged under 50, sample sizes were similar, with 7573 ELSA respondents and 8864 UKHLS respondents, giving a total sample size of 16,437. Given that socio-demographic missing data were negligible (Fig. 1) missing cases were removed at this stage. Details on missing biomarker data are discussed below.

**Table 1:** Sample characteristics.

	ELSA	UKHLS	Pooled sample
	n = 7573	n = 8864	n = 16,437
**Intersectional attributes**			
Age—mean (SD)	67.63 (9.36)	64.90 (9.94)	66.16 (9.77)
Women—% (n)	55.26 (4185)	54.22 (4806)	54.70 (8991)
BME—% (n)	2.93 (222)	3.34 (296)	3.15 (518)
Low education—% (n)	59.70 (4531)	56.62 (5019)	58.04 (9540)
Low income—% (n)	33.34 (2525)	33.37 (2968)	33.36 (5483)
Medium income—% (n)	33.25 (2518)	33.35 (2956)	33.30 (5474)
High income—% (n)	33.41 (2530)	33.28 (2950)	33.34 (5480)
**Biomarkers—mean (SD)**			
HbA1c (mmol/mol)	41.16 (8.36)	39.45 (8.64)	40.32 (8.54)
Missing—% (n)	24.85 (1882)	38.08 (3375)	31.98 (5257)
Cholesterol (mmol/L)	5.54 (1.18)	5.53 (1.22)	5.53 (1.20)
Missing—% (n)	23.95 (1814)	34.18 (3030)	29.47 (4844)
CRP (mg/L)	2.13 (1.92)	2.23 (2.03)	2.18 (1.98)
Missing—% (n)	28.28 (2142)	39.49 (3500)	34.32 (5642)
SBP (mm Hg)	132.21 (17.49)	131.43 (17.25)	131.81 (17.37)
Missing—% (n)	6.89 (522)	15.13 (1341)	11.33 (1863)
RHR (bpm)	66.49 (10.60)	68.45 (11.00)	67.50 (10.85)
Missing—% (n)	6.88 (521)	15.13 (1341)	11.33 (1862)
BMI (kg/m^2^)	28.30 (5.26)	28.55 (5.27)	28.43 (5.27)
Missing—% (n)	4.41 (334)	5.92 (525)	5.23 (859)

Ethics approval including for biomarker collection and prospective analysis was sought by the respective studies. Ethics approval was not necessary for the present study as it uses secondary data only.

### Intersectional variables

Gender, ethnicity, education and income were used to define 24 intersectional subgroups. Given the interest in intersecting disparities, sample size limitations meant it was necessary to use relatively coarse categorisations. In ELSA, participants were asked to which ethnic group they felt they belonged with seven options. In UKHLS, respondents were asked their ethnic group from 18 options. Ethnicity was dichotomised into White/Black and Minority Ethnic (BME). For ELSA this was defined by the category ‘white’ versus any other response, and for UKHLS the categories ‘white British/English/Scottish/Welsh/Northern Irish’, ‘Irish’, and ‘Any other white’, versus any other response. Whilst the BME category contains substantial ethnic heterogeneity, and is the subject on ongoing debate, we are interested in minority ethnic status as a key dimension of social and health inequality in the English context. We were also restricted by sample size, which for specific ethnic groups is too small to expect meaningful differences to be uncovered.

**Figure 2:**
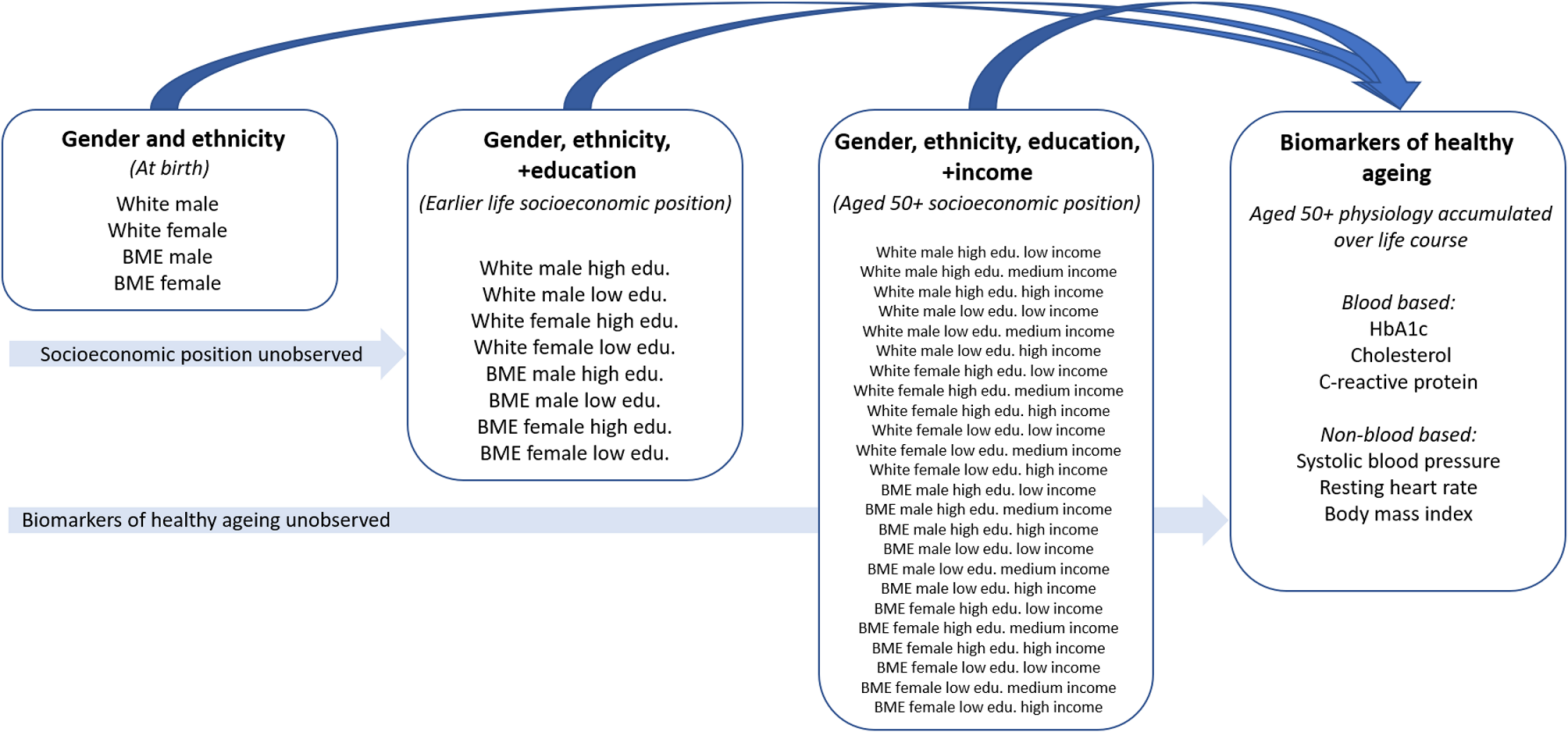
Conceptual framework.

To capture both earlier and later life SEP, we analysed both education and income. Education sets in motion a (non-determinant) trajectory of socioeconomic dis/advantage at a relatively early stage of the life course. Income at age 50 is a result of dis/advantage accumulated through an individual’s life. Further, in the English context, retirement income is strongly linked to pre-retirement SEP. Education and income are themselves outcomes of intersectional processes, but nonetheless are strongly associated with particular social positions and identities. We represent the conceptual framework underlying the analysis in Fig. 2. For both surveys, education was measured according to whether respondents had an A level (or NVQ lever 3) or higher. For income, ELSA collects information at the benefit unit level, which is a couple or single person and any dependent children. This is equivalised by adjusting for benefit unit size. In UKHLS, income was measured at the household level and equivalised using the OECD scale. Given that the sources and marginal utility of income change over the life course, we split income into tertiles to maximise comparability between the different intersections. Since in ELSA ages over 90 are collapsed into one category, the same procedure was applied to the UKHLS dataset. We controlled for age (including a squared term to capture non-linear effects, which healthy ageing biomarkers often exhibit^24^) as our aim is to map out health disparities in relation to socioeconomic and socio-demographic factors. Further, the extent to which biomarkers are risk factors for disease is age variant.

### Outcomes

Biomarkers were derived from bloods (HbA1c, total cholesterol, C-reactive protein (CRP)) and anthropometric/cardiovascular measures (systolic blood pressure (SBP), resting heart rate (RHR), body mass index (BMI)). HbA1c is a measure of blood glucose concentration over the past 2 to 3 months and is used to diagnose diabetes^25^. It is associated with diabetes complications and cardiovascular events and stroke, including in patients without diabetes^26^. Total cholesterol is a well-established risk factor for cardiovascular disease (CVD)^27^. CRP is a marker of inflammation thought to respond to cumulative social adversity and is a predictor of CVD^28^ and diabetes^29^. Systolic, rather than diastolic, blood pressure was analysed as it is a predictor of CVD^30^. Resting heart rate is an independent risk factor for CVD and all-cause mortality^31^ as well as metabolic syndrome^32^. BMI is risk factor for a range of diseases^33^. We did not adjust for medication use (and therefore disease) as this is part of the underlying causal pathway of interest i.e. biomarkers as indicators of healthy ageing and disease. We present unstandardised results as we are interested in the clinical significance of intersectional disparities but use standardised outcomes when estimating model fit statistics as these are scale-dependent. We standardised outcomes so that outcomes had a mean of 0 and a standard deviation of 1. Measurement error with biomarker outcomes is likely to be minimal given the standardised measurement procedures implemented in ELSA and UKHLS.

### Missing biomarker data

For blood measures, in ELSA 1882 (24.85%) respondents were missing values for HbA1c, 1814 (23.95%) for cholesterol and 2142 (28.28%) for CRP. 602 (7.95%) respondents refused to give a blood sample and 364 (4.81%) were not eligible due to a clotting/blood disorder. In 829 (10.95%) cases there were problems with measurement for example due to poor veins or an incomplete or non-reactive sample. In 328 cases CRP was > 10 mg/L, indicating recent infection, and was recoded as missing following best practice^34^. For non-blood measures, 522 (6.89%) respondents were missing values for SBP and 521 for RHR (6.88%). In 384 cases this was because they had engaged in an activity in the last 30 minutes that would affect their measures, with remaining cases due to invalid or incomplete measures. 334 (4.41%) respondents were missing values for BMI nearly all due to measurement issues.

In UKHLS, 3375 (38.08%) respondents were missing values for HbA1c, 3030 (34.18%) for cholesterol and 3500 for CRP (34.49%). 1380 (15.57%) respondents refused to give a blood sample, and 803 (9.06%) were ineligible for unspecified reasons. In 381 cases CRP was > 10 mg/L, and was recoded as missing. For non-blood measures, 1341 (15.13%) respondents were missing values for SBP and RHR due to 1130 (12.75%) engaging in an activity in the last 30 min that would affect their measures, with the remaining causes due to invalid/incomplete measures. 525 (5.92%) respondents were missing cases for BMI nearly all due to measurement issues.

In cross-sectional analysis, missing data do not introduce bias provided all variables associated with missingness are included as covariates, under a missing at random assumption^35^. Given the included socio-demographics are likely to be highly associated with missingness, we would expect their inclusion to minimise bias. As a sensitivity test, missing data were imputed using multiple imputation following best practice guidelines^35^. Chained equations were used, with all biomarkers to be imputed included in the model, and all analysis variables included as predictors, as well as auxiliary variables known to be associated with missingness as stated in the user guides for ELSA and UKHLS. We used the pooled data for imputation as auxiliary variables were available across both surveys: marital status (married/not married), household size, home ownership (owner/non-owner), long-standing illness, self-rated health (5 categories). We used long-standing illness as a substitute for limiting long-standing illness as only the former was available across both datasets. We were also unable to include government office region, also known to be associated with missingness, as it is anonymous in ELSA and therefore cannot be pooled. The number of imputations was set to 34—the % missing for the variable (CRP) with most missing data for the pooled sample. As the differences between the imputed and non-imputed data were non-existent or negligible (Supplementary Fig. 2), we proceed with complete case analysis (by outcome).

### Survey weights, clustering and stratification

As a sensitivity analysis, we followed guidelines on weighting data and accounting for clustering and stratification from the user guides of ELSA and UKHLS. For ELSA, the weight variable was w6bldwt for analysis of blood biomarkers and w6nurwt for non-blood biomarkers, and for UKHLS the respective variables were indbdub_xw and indnsub_xw. Since differences with unweighted data were small (Supplementary Fig. 3 and Supplementary Table 1), and it is not possible to weight pooled data due to different sample designs, we present the pooled unweighted data on the basis that the precision gained by pooling two national datasets is greater than that gained by analysing two sets of weighted data separately. Ethnicity was most sensitive to weighting, consistent with the other analyses in the paper. We discuss this in the limitations.

**Figure 3:**
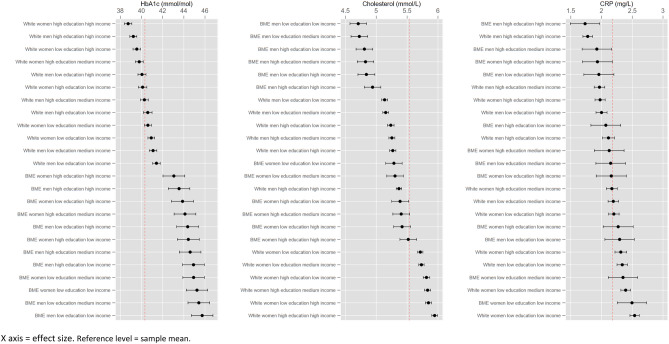
Intersectional disparities in blood biomarkers.

### Statistical analysis

We first use the multilevel analysis of individual heterogeneity and discriminatory accuracy (MAIHDA) method which is a novel multilevel approach developed to analyse intersectional inequalities^9,36^. MAIHDA has been used to study various types of health outcomes, including depression^13^, body mass index^11,14^, chronic obstructive pulmonary disease^15^, and opioid misuse^37^. This method involves defining a number of intersectional groups (or intersections) according to combinations of social attributes. These intersections are taken to be analogous to the types of contexts traditionally studied in multilevel models such as neighbourhoods or countries^37^. For example, one intersection may be BME women with high education and low income. Given this method is explicitly testing multiple hypotheses (the differences between multiple intersections), readers might be concerned about multiple testing. Jones et al.^38^ argue that shrinkage in MAIHDA models solves the problem of multiple testing, but we have shown in previous work^36^ that this is not always the case. However, this should be seen as a sign of more certainty in our null finding of no multiplicative effects: there were no significant effects even though the test applied was potentially too anti-conservative.

First, a null model is specified with individuals at level one nested within intersections at level two. This allows for estimating the extent to which the variance in an outcome is explained by differences across intersections versus differences within them via the intraclass correlation coefficient (ICC)—in the MAIHDA framework termed the variance partition coefficient (VPC). A high VPC suggests that intersections have substantially different mean levels of an outcome and that individuals are fairly similar within them, whereas a low VPC suggests that individuals differ substantially within intersectional groups, which have similar mean levels of a particular outcome^9^.

In the second step, main effects are added to the fixed part of the model. The VPC now represents the extent to which intersectional clustering is multiplicative. A reduction in the VPC to around 0% indicates that intersectional differences are fully explained by the main effects, and so are additive and not multiplicative. Given we find no evidence for multiplicative effects, the MAIHDA models offer no advantages over conventional models for estimating mean differences in outcomes between intersectional groups^36^, so we proceed with conventional linear regression analysis and use marginal effects to predict intersectional disparities, and we use MAIHDA to ascertain the discriminatory accuracy of intersectional clustering (i.e. the extent to which intersections are able to distinguish between different levels of a biomarker). The BIC values in Supplementary Table 2, which we use to assess the trade-off between model fit and complexity as is typically done when using MAIHDA^11^, confirm that linear regression models have no worse model fit than the multilevel main effects models.

**Figure 4:**
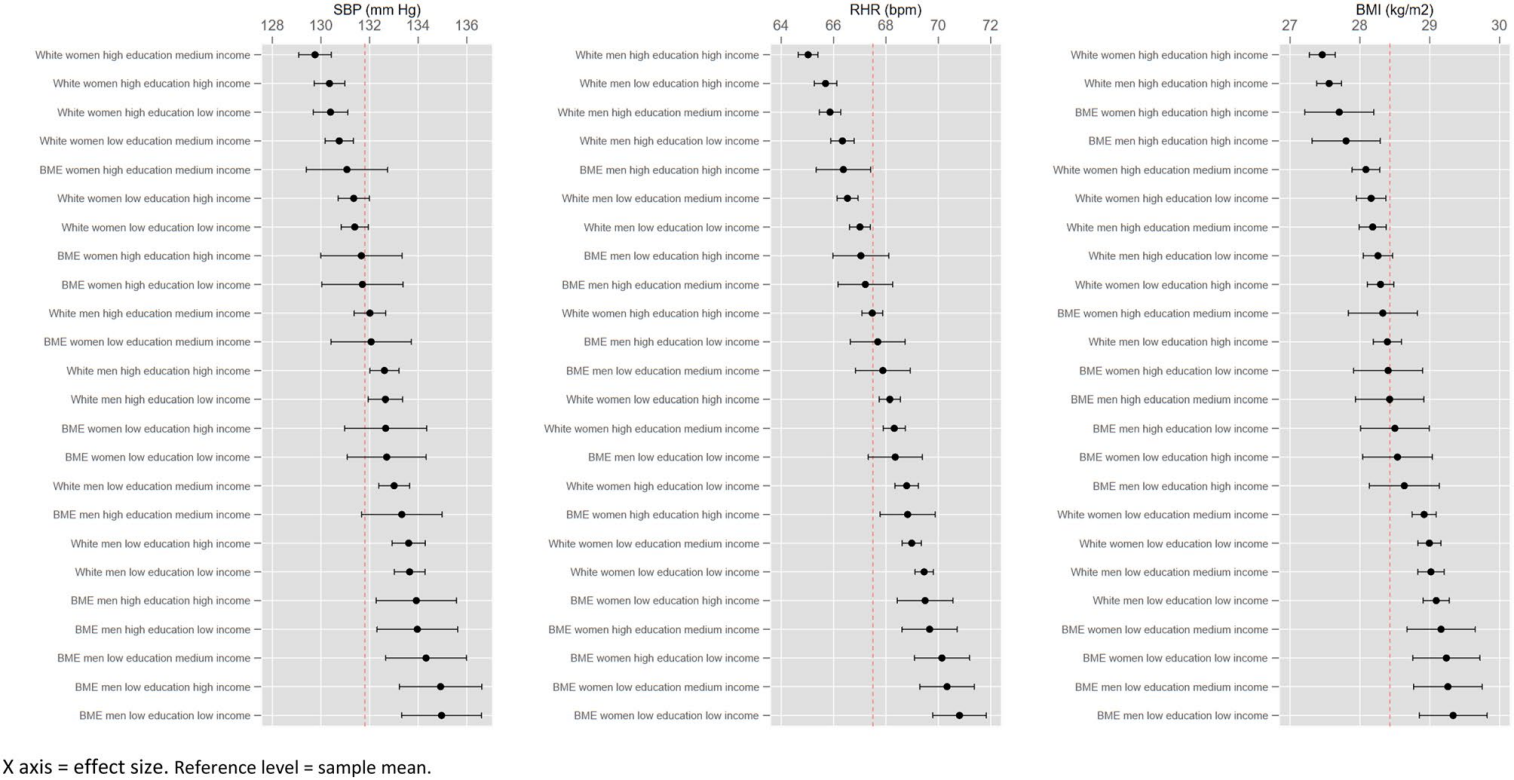
Intersectional disparities in non-blood biomarkers.

**Table 2:** Coefficient estimates from linear regression main effects models.

	HbA1c (mmol/mol)	Cholesterol (mmol/L)	CRP (mg/L)	SBP (mm Hg)	RHR (bpm)	BMI (kg/m^2^)
Women	− 0.49 (− 0.81 to − 0.17)	0.58 (0.54–0.62)	0.20 (0.13–0.28)	− 2.26 (− 2.82 to − 1.70)	2.45 (2.10–2.81)	− 0.10 (− 0.26–0.07)
BME	4.32 (3.33–5.30)	− 0.43 (− 0.56 to − 0.30)	− 0.04 (− 0.27–0.19)	1.31 (− 0.27–2.90)	1.35 (0.35–2.35)	0.24 (− 0.23–0.71)
Low education	0.82 (0.49–1.15)	− 0.10 (− 0.14 to − 0.06)	0.22 (0.15–0.30)	1.00 (0.40–1.59)	0.67 (0.29–1.04)	0.83 (0.66–1.01)
Low income	1.37 (0.96–1.78)	− 0.13 (− 0.19 to − 0.08)	0.34 (0.24–0.43)	0.04 (− 0.69–0.77)	1.31 (0.85–1.77)	0.70 (0.48–0.91)
Medium income	1.05 (0.66–1.44)	− 0.11 (− 0.17 to − 0.06)	0.19 (0.10–0.28)	− 0.60 (− 1.29–0.10)	0.84 (0.40–1.28)	0.62 (0.42–0.83)
Age	0.64 (0.44–0.85)	0.02 (− 0.00–0.05)	− 0.04 (− 0.08–0.01)	1.27 (0.92–1.63)	− 0.54 (− 0.77– − 0.32)	0.28 (0.17–0.39)
Age squared	− .004 (− .005 to − .002)	− .000 (− .001 to − .000)	.000 (− .000 –.001)	− .007 (− .010 to − .005)	.004 (.002–.005)	− .002 (− .003 to − .002)
BIC	31,377	31,645	30,495	40,881	41,114	44,008
n	11,180	11,593	10,795	14,574	14,575	15,578

BIC values pertain to standardised outcomes.

MAIHDA models were estimated using runmlwin^39^ v2.36 (MLwiN v3.04^40^), called from Stata v14.0. Linear regression models were estimated directly in Stata and marginal effects were calculated using the margins command. Given that MAIHDA models are often estimated using the Markov Chain Monte Carlo (MCMC) method^13–15,37^ we tested whether the lack of multiplicative effects found was due to our use of IGLS estimation. Further, MAIHDA models often use age categories to define the intersectional subgroups^9,13–15,37^, so we also tested whether this strategy would result in multiplicative effects being found, by including age in 10 years bands to define intersectional subgroups. Supplementary Table 3 shows that this made a negligible difference to the lack of multiplicative effects we found, with ICC values for main effects models of ~ 0–1.5%. For this supplementary analysis, MCMC estimation was based on IGLS initialisation values (burn-in length 5000 iterations; monitoring chain length 50,000 iterations). MCMC models demonstrated marginally higher VPC values but differences were minimal. The inclusion of age categories to define the intersections made no difference to the multiplicative effects found. Stata .do files are included for replication of all analyses in the supplementary materials.

**Table 3:**
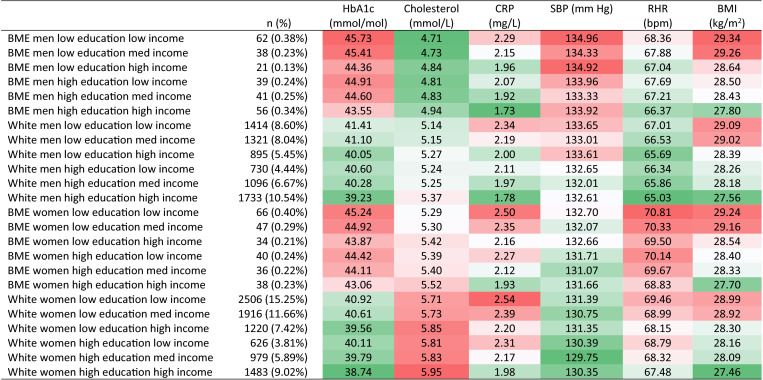
Disparities in biomarkers across intersections.

Shading illustrates effect size; range of shading equivalent to range of intersectional differences by biomarker. Opaque green indicates low relative biomarker level; opaque red indicates high relative biomarker level.

## Results

ELSA and UKHLS were well-matched on nearly all variables (Table 1). In terms of biomarker outcomes, mean values were above commonly accepted clinical cut-points for cholesterol (5 mmol/L^25^, sample mean 5.53) and SBP (120 mm Hg^41^, sample mean 131.81). HbA1c was below the cut-point of 48 mmol/mol for diagnosis of diabetes^25^ (sample mean 40.32), CRP was below the suggested cut-point of > 3 mg/L for metabolic syndrome^29^ (sample mean 2.18); and RHR was below 120 bpm, which is considered high^42^ (sample mean 67.50). The sample mean for BMI (28.43 kg/m^2^) was above the cut-point for overweight but not obese. The intersectional distribution is shown in Supplementary Table 4.

### Multilevel intersectional models

The null multilevel intersectional models showed small overall intersectional variation (Supplementary Table 2)—around 1 to 2%—with the exception of HbA1c (5.64%) and cholesterol (9.18%). Thus 1–3% of the variance in CRP, SBP, RHR and BMI is at the intersectional level, and the majority of variance is at the individual level. This suggests that there is substantial heterogeneity within, and overlap between, intersections for these biomarkers, and they are relatively poor at discriminating between individuals in terms of their biomarker levels (less so for HbA1c and cholesterol). Once the main effects were added to the fixed part of the models, the ICC reduced to ~ 0% in each case, meaning there was no evidence of multiplicative intersectional effects.

### Multiple regression models

The multiple regression models suggest additive intersectional effects, with the various social attributes mostly having an independent effect on biomarker outcomes (Table 2). The mixed direction of the effects presents a complex picture regarding disparities in objective measures of healthy ageing, suggesting that there may be variegated processes and mechanisms underlying these disparities. For comparison, standardised results are given in Supplementary Table 5.

Controlling for age, women had lower HbA1c and SBP, but higher cholesterol, CRP, and RHR than men. While the BME category had higher HbA1c, SBP and RHR, they had lower cholesterol compared with the White category, and there was no difference for other outcomes. Low education was associated with elevated biomarker levels except cholesterol where the opposite was the case. For income, HbA1c, CRP and RHR followed a gradient pattern where lower income was associated with elevated biomarker levels. This was reversed for cholesterol, and for SBP medium income had the lowest figure. For BMI, those with high income were distinct in having lower levels.

While these differences are informative of the effects of social attributes across the whole sample, they mask relatively large subgroup differences, and the mixed relationships suggest intersectional patterning, where the factors in the regression add together to produce multiple (dis)advantages. We therefore plotted predicted results by intersectional subgroup (Figs. 3 and 4), showing that disparities between subgroups are much wider than conventional multiple regression results convey.

### Predicted intersectional disparities

In terms of blood-based biomarkers, intersections ranged in mean predicted HbA1c from 38.73–45.73 mmol/mol, with a sample mean of 40.32 mmol/mol. The effect of ethnicity on HbA1c was apparent, and there was a difference of around 2.6 mmol/mol within both ethnic categories, according to the other intersectional attributes. The BME intersection with the lowest HbA1c still had a value around 1.5 mmol/mol higher than the white intersection with the highest HbA1c. Intersections ranged in predicted mean cholesterol from 4.71–5.95 mmol/L. BME male intersections had the lowest cholesterol, with small education/income variation within this group, whilst white female intersections had the highest, again with this group mostly homogenous. Intersections ranged in predicted CRP from 1.73–2.54 mg/L. Those with high income and education had the lowest levels, especially for men, regardless of ethnicity. Conversely the highest levels were seen amongst women in the lowest income and education categories.

For non-blood biomarkers, intersections ranged in predicted SBP from 129.75–134.97 mm Hg, with a sample mean of 131.81 mm Hg. BME men, and especially those with low education had the highest SBP. White men with low education had a similar SBP to BME men with high education. White women had low SBP, and the lowest levels were found in those with high education. White men with high education and BME women with low education had around the sample mean. Intersections ranged in predicted RHR from 65.03–70.81 bpm. Despite the strong gender main effect, there was overlap between some male and female intersections, with BME men with low education and white women with high education near the sample mean of 67.50 bpm. White male intersections with high incomes had the lowest RHR while BME female intersections with low or medium incomes had the highest RHR. Intersections ranged in predicted BMI from 27.46–29.34 kg/m^2^, with a sample mean of 28.43 kg/m^2^. The effect of low or medium income was apparent across all gender/ethnic categories, and BME men and women with low or medium incomes had the highest BMI predictions. Conversely, those with high incomes in particular had the lowest BMIs, and again this was mostly invariant according to other categories.

Table 3 summarises differences across biomarkers for each intersectional group, showing that intersections are rarely dis/advantaged according to all biomarkers—though some are more than others. For example, white men typically had low levels across multiple biomarkers but nonetheless white men with low education had elevated SBP and BMI. White men with high education had reduced HbA1c, CRP, RHR, and BMI, but not cholesterol or SBP. For the equivalent white women high cholesterol was clearly elevated. BME men had high HbA1c, SBP and BMI (except those with high education and income) but low cholesterol. BME women had elevated levels across a number of biomarkers—HbA1c, CRP, RHR, and BMI (except those with high education and income), but did not have lower cholesterol like their male counterparts. Overall, education and income can to some extent counteract gender and ethnic disparities in biomarker measures, but not completely.

These heterogeneous patterns open up the possibility of biomarker health ‘profiles’ across different intersectional groups (which must nonetheless be viewed in the context of the majority of heterogeneity existing within intersections rather than between them). This suggests there may be differential intersectional drivers and healthy and functional ageing outcomes related to these profiles.

## Discussion

For the first time, we have intersectionally ‘mapped out’ the main social disparities in key biomarkers of healthy ageing using nationally representative English data. We found no evidence of multiplicative intersectional effects, consistent with other MAIHDA analyses which have generally found no or negligible effects in a range of health outcomes^13–15,37^. We uncovered intersectional disparities both in terms of the intersectional range, as well as intersectional patterning, as a result of the additive (or layered) effects of social attributes. The intersections nonetheless exhibited low discriminatory accuracy. Methodologically, our analysis suggests that although the MAIHDA method is useful for distinguishing between additive and multiplicative effects and the discriminatory accuracy of intersectional subgroups, conventional main effects regression is a more parsimonious way to explore intersectional disparities in the absence of multiplicative effects.

The research and policy significance of additive vs multiplicative effects is a key theme in the intersectionality literature^19,43–45^. We agree with the position that actual intersectional disparities (accounting for any potential interactions if necessary) are a key tenet of the intersectionality framework^19^. These disparities are more important than their statistical constitution^46^, and are relevant to policy interest in targeting and evaluating policies and interventions in relation to those facing multiple disadvantages^47^. Further, the social uniqueness of intersectional positions/identities will not necessarily translate into multiplicative effects^13^.

Whilst further work is needed to understand whether intersectional disparities translate to differential risk of health events, existing work suggests that the mean differences in biomarker levels across intersections represent clinically important differences. For example, mean intersectional HbA1c varied from 38.82–45.72 mmol/mol, approximating a 5% increase in cardiovascular mortality in those with no known diabetes^48^ and a 17% increased risk of cardiovascular events in those with type 2 diabetes^49^. The intersectional range of 1 mmol/L in total cholesterol is associated with a ~ 20% increase in coronary heart disease (CHD) in women and a ~ 24% increase for men^50^. Mean intersectional CRP varied from 1.76–2.52 mg/L, which is associated with a ~ 50% increased risk of CHD and ~ 20% risk in vascular death^51^. The intersectional range of ~ 5 mm Hg in SBP is associated with a ~ 20% increase in the risk of stroke death and ~ 15% increase in risk of death from ischaemic heart disease and other vascular causes^52^. The intersectional range in RHR of 6 bpm is associated with an increased risk of CHD of ~ 4%, CVD of ~ 8%, and all-cause mortality of ~ 9%^53^. Finally the intersectional range in BMI of ~ 2 kg/m^2^ is associated with an increase in the hazard ratio for mortality of around 1.08; put another way, the intersectional level of just under 30 kg/m^2^ is associated with living around 4 years less compared to those with a BMI of < 25^33^. These comparisons are approximate given differing populations and confounder adjustment but nonetheless are indicative of the potential implications of wide-ranging intersectional disparities in biomarkers.

The main effects driving the intersectional patterning were approximately consistent with previous studies. For example, a study using UKHLS data found very similar effects for gender and income for the six biomarkers analysed here, despite also including younger adults^54^. Studies on the relationship between ethnicity and biomarkers of healthy ageing seem to be lacking in the extant literature. We found that intersectional disparities according to gender, ethnicity, education and income did not follow a simple pattern but were different for different biomarkers. Consequently, different intersectional subgroups have different ‘biomarker profiles’, and although some intersections have a greater number of reduced or elevated biomarkers than others, no intersection has reduced or elevated levels of all biomarkers. Similarly, our findings also highlight that it is important to move away from mean differences between e.g. men and women, and between White and BME groups given the heterogeneity within these categories according to other social attributes. For example, we found evidence of compensatory mechanisms such that elevated ethnic minority HbA1c was somewhat but not completely compensated by socioeconomic advantage. For SBP, we found that the most advantaged men and the most disadvantaged women have approximately the same SBP, despite the overall strong gender effect.

An important caveat to these findings emerging from the use of the MAIHDA is that the intersections exhibited low discriminatory accuracy. Most heterogeneity in biomarker outcomes exists within rather than between intersectional subgroups, consistent with the picture emerging of MAIHDA analyses of various outcomes^11,14,15,37^. In other words, intersections cannot discern which particular person is healthy or sick. This speaks to emerging findings from ‘precision medicine’^55^, where it is becoming evident that current statistical methods are unable to predict the health of any one individual with any reasonable degree of accuracy. Caution is therefore required in targeting interventions or policies, not only due to matters of efficiency, but also given the risk of stigmatising people who are assumed to have a particular level of health or illness by virtue of their social attributes^18^. Nonetheless action on particular intersections could still have important population health effects if they are effective at shifting the means for those subgroups.

It remains to be seen whether intersections have better discriminatory accuracy with regards to other factors the theory suggests are patterned intersectionally such as differences in social discrimination, lived experience, or being subject to differential institutional or policy processes. In addition, differential life course drivers e.g. in terms of the social, political or geographical context in relation to an individual’s life trajectory, may be implicated in intersectional patterning, providing clues on how to design policies and interventions to address this^56^. However in the absence of this evidence caution is required in intersectionally targeting/tailoring interventions or policies at particular subgroups. An alternative more social epidemiologically-oriented research goal might be to uncover the social factors driving the individual distribution of risk^9^, in which case discriminatory accuracy matters less. Indeed, researchers are now turning towards answering the question, for example by investigating whether discrimination mediates intersectional patterning^18^.

In presenting our findings, we have deliberately used the term disparities to denote that the differences demonstrated do not necessarily represent inequalities because they may to some extent reflect natural underlying physiological differences. For example, men and women have different anthropometric profiles and so their different biomarker levels might be considered ‘normal’. Similarly, there is some debate whether there should be ethnic-specific cut-points in HbA1c for diagnosing diabetes^57^. Given that we analyse a range of biomarkers and social attributes it would be unfeasible to take into account the difference between levels of biomarkers (or biomarker profiles) and consequences for healthy and functional ageing in the current analysis. Instead we regard this as an important focus of future work. A potentially interesting interim step might be to investigate the pathways between biological processes and rates of diagnosed diseases, which might suggest areas of over or under-diagnosis, or point towards the social processes and mechanisms such as help-seeking and diagnosis mediating this relationship.

Our study has a number of strengths. Pooling biomarker data from ELSA and UKHLS has allowed for a granular analysis of intersectional disparities in biomarkers of healthy ageing and disease across England. Whilst many health inequalities studies control for socio-demographics, for example controlling for socioeconomic status in explaining ethnic differences, we specifically focus on subgroup disparities in order to examine their co-constitution by multiple social attributes.

Nonetheless, as with all studies ours has limitations. We did not take into account the use of medications which affect biomarker measures because medication use is confounded with diagnosis and severity, which are ultimately of interest in mapping out biomarker disparities. It is likely that intersectional differences vary according to whether people are diagnosed and on medication or not and this should be explored in further work. There may also be intersectional bias e.g. in terms of non-response which influences the patterns we found. Some intersectional effects may be due to how the meaning of some categories changes according to others e.g. educational qualifications and age. Ethnicity is notoriously difficult to measure in surveys, with a range of different meanings and constructs that impact on the way it is recorded. We found some differences between ELSA and UKHLS in terms of ethnic differences in biomarkers, including in sensitivity analyses. Nonetheless, we consider our results as broadly indicative of intersectional inequalities, but they should be seen as exploratory and in need of further investigation using larger samples, ideally with high quality ethnicity data. Our study would likely have been able to uncover greater ethnic heterogeneity using such data for example by focussing on particular ethnic groups. We cannot generalise out findings beyond the English setting. Given the minimal measurement error with biomarker outcomes, an interesting area of future research would be to see whether intersectional inequalities vary between different geospatial contexts.

## Conclusion

We present a complex picture of disparities in healthy ageing biomarkers to counter dominant simplistic narratives of health inequalities according to single or at most a limited number of social attributes e.g. SEP or ethnicity. Our results have a number of important implications. They show that certain intersections have particularly reduced, or elevated levels of particular biomarkers of healthy ageing and chronic disease, which should be taken into account in inequity policy. However, our results also show that intersections have substantial heterogeneity i.e. that they are relatively poor at discriminating who has an elevated or reduced level of any particular biomarker. This suggests that rather than using intersections to target interventions, which may be inefficient, policies might instead aim to affect the underlying social determinants of health that are responsible for overall intersectional differences. Our results also suggest that people who are typically disadvantaged according to multiple social attributes e.g. gender, income and ethnicity, are not always disadvantaged according to all health measures. Rather, a more nuanced picture emerges, suggesting intersectional groups exhibit a mixture of both protective as well as risk factors for illness.

By opening up a granular view of inequalities, an intersectionality research agenda offers opportunities to unpack how the dynamics of power and social determinants might interact to drive variegated outcomes. This presents new puzzles and challenges on how and why biomarkers might translate into health and functional outcomes differently for different subgroups and the life course processes that lead to such differences. Such innovation may be helpful in tackling the huge and growing burden of chronic disease.

## Supplementary information

Supplementary information 1.

Supplementary information 2.

## Data Availability

The datasets analysed in the current study are freely available from the UK Data Archive.
