# Supplementary material for: Mapping intersectional inequalities in biomarkers of healthy ageing and chronic disease in older English adults
Source: Sci Rep. 2020 Aug 11;10:13522. doi: 10.1038/s41598-020-69934-8 (PMC7419497; doi:10.1038/s41598-020-69934-8)
Supplement: Supplementary file 1 — Supplementary information 1. [file 41598_2020_69934_MOESM1_ESM.docx]

Mapping intersectional inequalities in biomarkers of healthy ageing and chronic disease in older English adults

Dr Daniel Holman, Department of Sociological Studies, University of Sheffield, UK* ([daniel.holman@sheffield.ac.uk](mailto:daniel.holman@sheffield.ac.uk))

Professor Sarah Salway, Department of Sociological Studies, University of Sheffield, UK

Dr Andrew Bell, Sheffield Methods Institute, University of Sheffield, UK

* Corresponding author

**Supplementary Figure 1 – Gender, ethnic, education and income inequalities in healthy ageing biomarkers – ELSA and UKHLS**


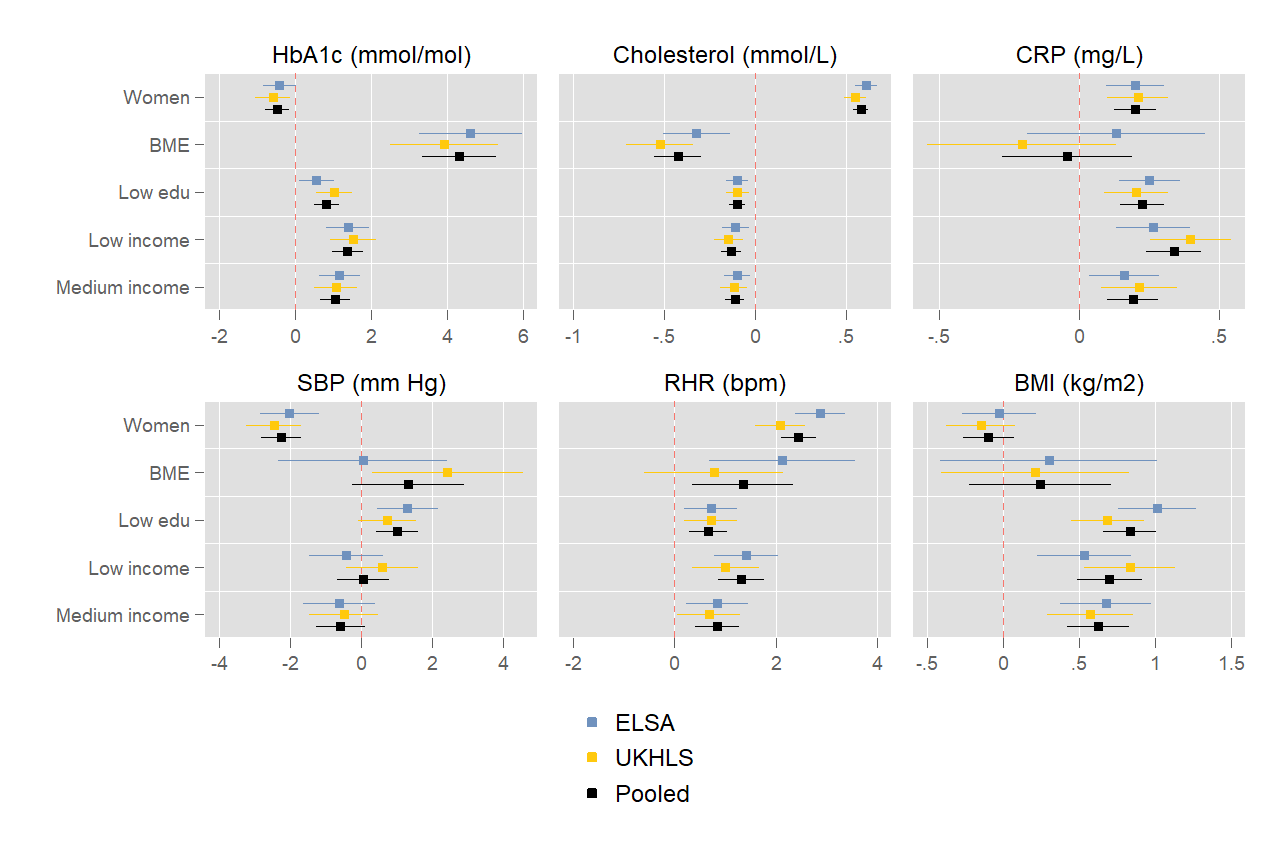
X axis = effect size. Bars represent 95% CIs.

**Supplementary Figure 2 – Gender, ethnic, education and income inequalities in healthy ageing biomarkers (non-imputed and imputed data) – ELSA and UKHLS**


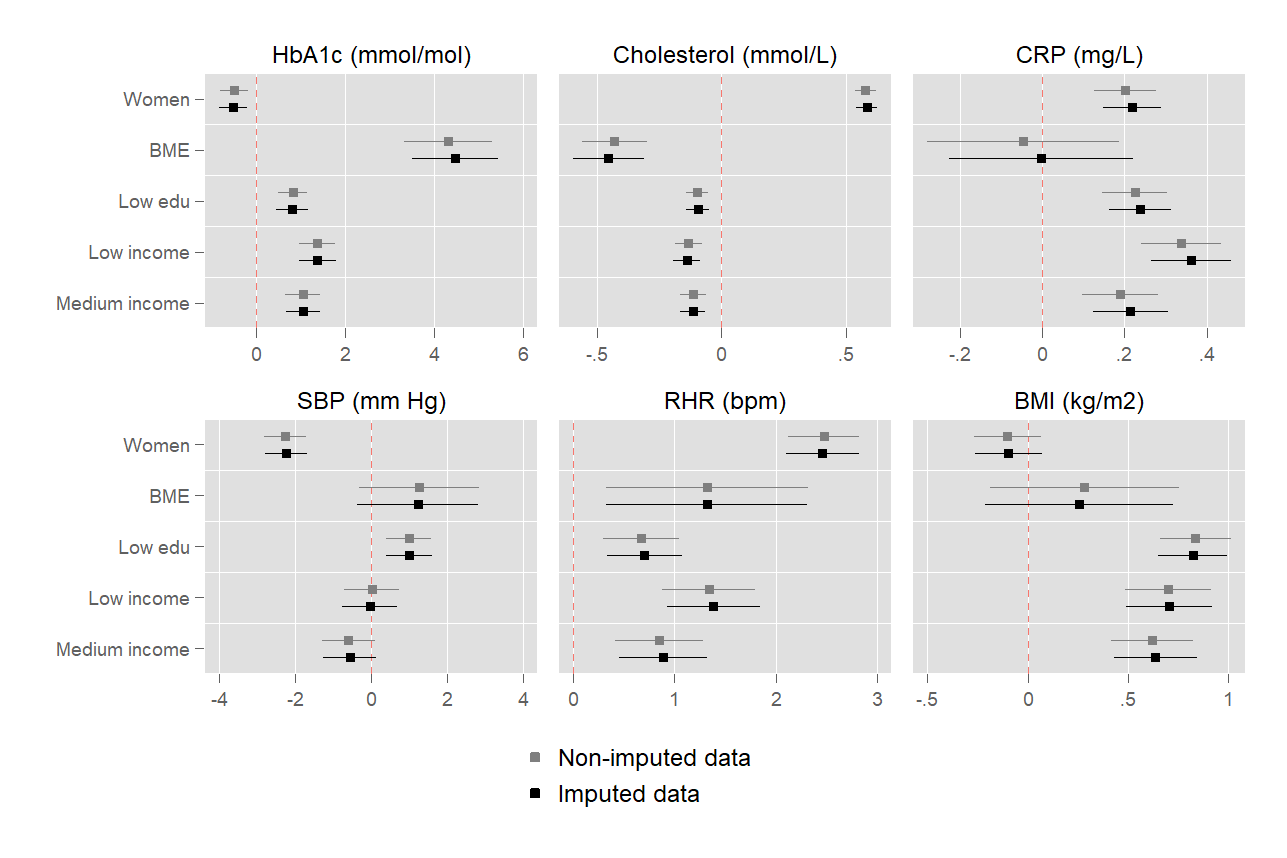


X axis = effect size. Bars represent 95% CIs.

**Supplementary Figure 3 – Gender, ethnic, education and income inequalities in healthy ageing biomarkers (unweighted and weighted data) – ELSA and UKHLS**


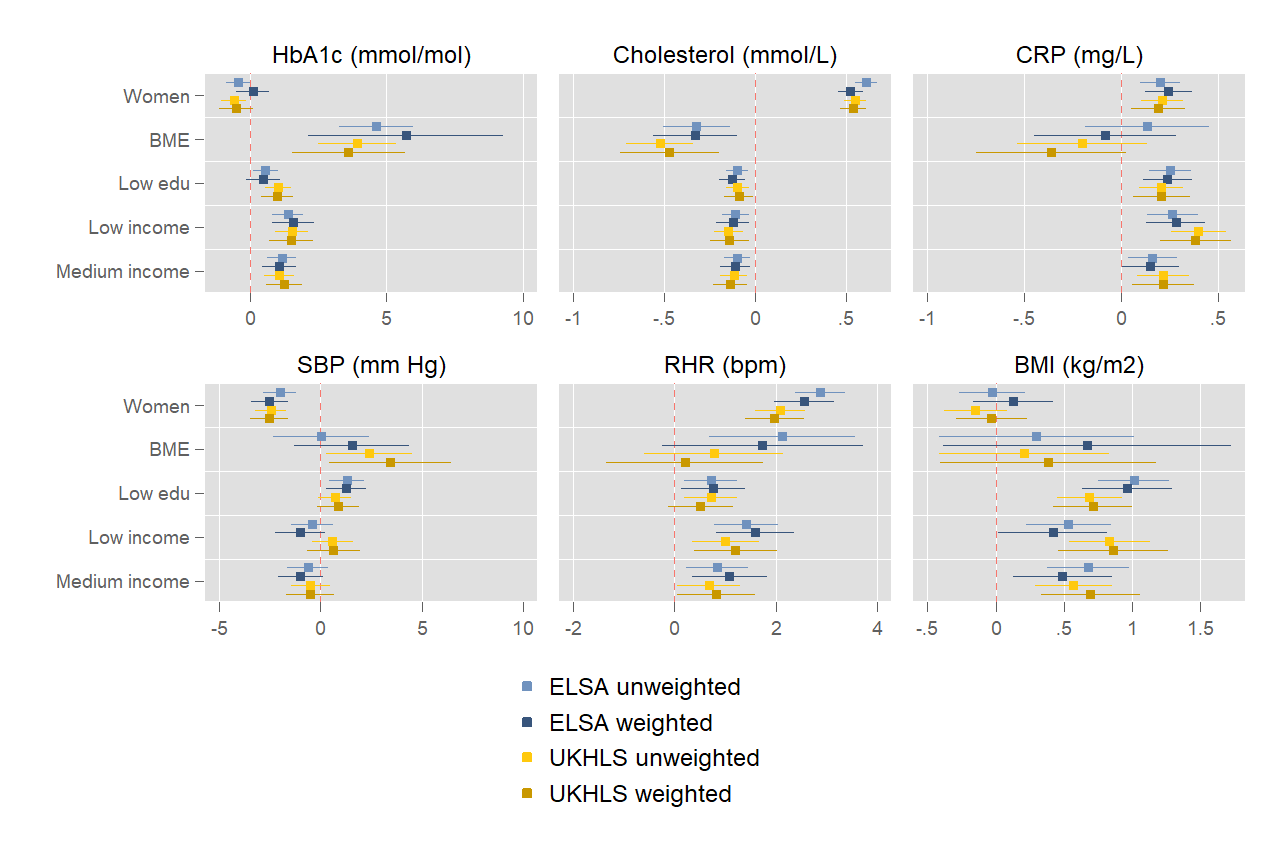
X axis = effect size. Bars represent 95% CIs.

**Supplementary Table 1 – Sample characteristics (weighted data)**

|  | ELSA | UKHLS |
| --- | --- | --- |
|  | n=7,530 | n=7,293 |
| Intersectional attributes |  |  |
| Age – mean (SD) | 65.44 (10.50) | 64.88 (10.33) |
| Women – % (n) | 52.41 (3,947) | 54.28 (3,958) |
| BME – % (n) | 5.22 (393) | 4.59 (335) |
| Low education – % (n) | 61.39 (4,623) | 58.12 (4,238) |
| Low income – % (n) | 33.86 (2,550) | 34.64 (2,526) |
| Medium income – % (n) | 32.97 (2,483) | 33.20 (2,421) |
| High income – % (n) | 33.17 (2,498) | 32.17 (23.46) |
| Biomarkers – mean (SD) |  |  |
| HbA1c (mmol/mol) | 41.27 (8.81) | 39.67 (8.93) |
| Missing – % (n) | 24.94 (1,878) | 42.22 (3,079) |
| Cholesterol (mmol/L) | 5.52 (1.17) | 5.50 (1.23) |
| Missing – % (n) | 24.05 (1,811) | 38.31 (2,794) |
| CRP (mg/L) | 2.15 (1.94) | 2.29 (2.06) |
| Missing – % (n) | 28.73 (2,164) | 43.51 (3,173) |
| SBP (mm Hg) | 131.44 (17.29) | 131.73 (17.36) |
| Missing – % (n) | 7.68 (578) | 15.44 (1,126) |
| RHR (bpm) | 66.83 (10.68) | 68.53 (10.99) |
| Missing – % (n) | 7.66 (577) | 15.44 (1,126) |
| BMI (kg/m^2^) | 28.43 (5.36) | 28.58 (5.34) |
| Missing – % (n) | 4.36 (328) | 5.88 (429) |

**Supplementary Table 2 – Summary of multilevel and linear regression models**

|  |  | Null model | | Main effects model | | Linear regression model |
| --- | --- | --- | --- | --- | --- | --- |
|  | n | BIC | ICC/VPC | BIC | ICC/VPC | BIC |
| HbA1c | 11,180 | 31417 | 5.64% | 31386 | 0.00% | 31377 |
| Cholesterol | 11,593 | 31680 | 9.18% | 31655 | 0.00% | 31645 |
| CRP | 10,795 | 30502 | 1.01% | 30504 | 0.00% | 30495 |
| SBP | 14,574 | 40870 | 0.61% | 40895 | 0.09% | 40881 |
| RHR | 14,575 | 41128 | 1.68% | 41133 | 0.01% | 41114 |
| BMI | 15,578 | 43993 | 1.63% | 44024 | 0.10% | 44008 |

IGLS estimation.
Outcomes are standardised.
All models control for age and age squared.
Level 2 = 26 intersectional subgroups defined by categories of gender, ethnicity, education and income.

**Supplementary Table 3 – Summary of multilevel models including age-defined intersections**

|  |  | Null model | | | | Main effects model | | | |
| --- | --- | --- | --- | --- | --- | --- | --- | --- | --- |
|  |  | IGLS estimation | | MCMC estimation | | IGLS estimation | | MCMC estimation | |
|  | n | BIC | ICC/VPC | DIC | ICC/VPC | BIC | ICC/VPC | DIC | ICC/VPC |
| HbA1c | 11,180 | 31499 | 3.94% | 31410 | 4.26% | 31454 | 0.05% | 31370 | 0.21% |
| Cholesterol | 11,593 | 31727 | 12.86% | 31578 | 13.36% | 31661 | 0.61% | 31556 | 0.89% |
| CRP | 10,795 | 30523 | 1.66% | 30466 | 1.78% | 30516 | 0.00% | 30444 | 0.16% |
| SBP | 14,574 | 40899 | 4.60% | 40791 | 4.85% | 40906 | 1.00% | 40782 | 1.37% |
| RHR | 14,575 | 41161 | 2.69% | 41076 | 2.84% | 41146 | 0.02% | 41057 | 0.23% |
| BMI | 15,578 | 44052 | 2.01% | 43976 | 2.15% | 44059 | 0.22% | 43962 | 0.38% |

With standardised outcomes.
All models control for age and age squared.
Level 2 = 87 intersectional subgroups defined by categories of age (10 years) gender, ethnicity, education and income.

**Supplementary Table 4 – Intersectional subgroups used in analysis**

| Intersections – n (%) | ELSA  n=7,573 | UKHLS  n=8,864 | Pooled sample  n=16,437 |
| --- | --- | --- | --- |
| BME men low education low income | 30 (0.40%) | 32 (0.36%) | 62 (0.38%) |
| BME men low education medium income | 18 (0.24%) | 20 (0.23%) | 38 (0.23%) |
| BME men low education high income | 4 (0.05%) | 17 (0.19%) | 21 (0.13%) |
| BME men high education low income | 17 (0.22%) | 22 (0.25%) | 39 (0.24%) |
| BME men high education medium income | 18 (0.24%) | 23 (0.26%) | 41 (0.25%) |
| BME men high education high income | 23 (0.30%) | 33 (0.37%) | 56 (0.34%) |
| White men low education low income | 620 (8.19%) | 794 (8.96%) | 1,414 (8.60%) |
| White men low education medium income | 646 (8.53%) | 675 (7.62%) | 1,321 (8.04%) |
| White men low education high income | 437 (5.77%) | 458 (5.17%) | 895 (5.45%) |
| White men high education low income | 290 (3.83%) | 440 (4.96%) | 730 (4.44%) |
| White men high education medium income | 484 (6.39%) | 612 (6.90%) | 1,096 (6.67%) |
| White men high education high income | 801 (10.58%) | 932 (10.51%) | 1,733 (10.54%) |
| BME women low education low income | 32 (0.42%) | 34 (0.38%) | 66 (0.40%) |
| BME women low education medium income | 18 (0.24%) | 29 (0.33%) | 47 (0.29%) |
| BME women low education high income | 13 (0.17%) | 21 (0.24%) | 34 (0.21%) |
| BME women high education low income | 20 (0.26%) | 20 (0.23%) | 40 (0.24%) |
| BME women high education medium income | 14 (0.18%) | 22 (0.25%) | 36 (0.22%) |
| BME women high education high income | 15 (0.20%) | 23 (0.26%) | 38 (0.23%) |
| White women low education low income | 1,222 (16.14%) | 1,284 (14.49%) | 2,506 (15.25%) |
| White women low education medium income | 898 (11.86%) | 1,018 (11.48%) | 1,916 (11.66%) |
| White women low education high income | 583 (7.70%) | 637 (7.19%) | 1,220 (7.42%) |
| White women high education low income | 294 (3.88%) | 332 (3.75%) | 626 (3.81%) |
| White women high education medium income | 422 (5.57%) | 557 (6.28%) | 979 (5.89%) |
| White women high education high income | 654 (8.64%) | 829 (9.35%) | 1,483 (9.02%) |

**Supplementary Table 5 – Coefficient estimates from linear regression main effects models with standardised outcomes**

|  | HbA1c (mmol/mol) | Cholesterol (mmol/L) | CRP (mg/L) | SBP (mm Hg) | RHR (bpm) | BMI (kg/m^2^) |
| --- | --- | --- | --- | --- | --- | --- |
| Women | -0.06 (-0.09- -0.02) | 0.48 (0.45-0.52) | 0.10 (0.06-0.14) | -0.13 (-0.16- -0.10) | 0.23 (0.19-0.26) | -0.02 (-0.05-0.01) |
| BME | 0.51 (0.39-0.62) | -0.36 (-0.46- -0.25) | -0.02 (-0.14-0.10) | 0.08 (-0.02-0.17) | 0.12 (0.03-0.22) | 0.05 (-0.04-0.14) |
| Low education | 0.10 (0.06-0.13) | -0.08 (-0.12- -0.05) | 0.11 (0.07-0.15) | 0.06 (0.02-0.09) | 0.06 (0.03-0.10) | 0.16 (0.12-0.19) |
| Low income | 0.16 (0.11-0.21) | -0.11 (-0.16- -0.06) | 0.17 (0.12-0.22) | 0.00 (-0.04-0.04) | 0.12 (0.08-0.16) | 0.13 (0.09-0.17) |
| Medium income | 0.12 (0.08-0.17) | -0.10 (-0.14- -0.05) | 0.10 (0.05-0.14) | -0.03 (-0.07-0.01) | 0.08 (0.04-0.12) | 0.12 (0.89-0.16) |
| Age | 0.08 (0.05-0.10) | 0.02 (-0.00-0.04) | -0.02 (-0.04-0.01) | 0.07 (0.05-0.09) | -0.05 (-0.07- -0.03) | 0.05 (0.03-0.07) |
| Age squared | -.000 (-.001- -.000) | -.000 (-.000- -.000) | .000 (.000-.000) | -.000 (-.001- -.000) | -.000 (.000-.000) | -.000 (-.000- -.000) |
| BIC | 31377 | 31645 | 30495 | 40881 | 41114 | 44008 |
| n | 11,180 | 11,593 | 10,795 | 14,574 | 14,575 | 15,578 |
