# Supplementary material for: Mapping intersectional inequalities in biomarkers of healthy ageing and chronic disease in older English adults
Source: Sci Rep. 2020 Aug 11;10:13522. doi: 10.1038/s41598-020-69934-8 (PMC7419497; doi:10.1038/s41598-020-69934-8)
Supplement: Supplementary file 2 — Supplementary information 2. [file 41598_2020_69934_MOESM2_ESM.docx]

Mapping intersectional inequalities in biomarkers of healthy ageing and chronic disease in older English adults

Stata .do files for replication

*Note: ELSA and UKHLS data from UK Data Archive required. Edit directory paths as required.*

**ELSA data preparation**

cd "${mystart}\.do files for NSR"

use "${mystart}\.do files for NSR\ELSA\UKDA-5050-stata\stata\stata11_se\wave_6_elsa_nurse_data_v2.dta", clear

keep if finstat==1 | finstat==7 | finstat==14 | finstat==25

gen refusedblood=0

foreach var of varlist refbscdi refbscne refbscte refbscil refbschi refbscot {

replace refusedblood=1 if `var'==1

}

gen innursewave=1

recode indager -7=90

drop if indager <50

keep idauniq hscrp hba1c pulse1 pulse2 pulse3 consubno w6nurwt w6bldwt chol SYSVAL BMIVAL WSTVAL hscrp innursewave refusedblood WSTOKB samdifno difbpcno clotb respwh BSOUTC BMIOK

save elsaw6biomarkers, replace

use "${mystart}\.do files for NSR\ELSA\UKDA-5050-stata\stata\stata11_se\wave_6_ifs_derived_variables.dta", clear

keep idauniq edqual

save elsaw6ifsedu, replace

use "${mystart}\.do files for NSR\ELSA\UKDA-5050-stata\stata\stata11_se\wave_6_financial_derived_variables.dta", clear

keep idauniq eqtotinc_bu_s

save elsaw6income, replace

use "${mystart}\.do files for NSR\ELSA\UKDA-5050-stata\stata\stata11_se\wave_6_elsa_data_v2.dta", clear

keep idauniq GOR Heill Helim idahhw6

save elsaw6gor, replace

use "${mystart}\.do files for NSR\ELSA\UKDA-5050-stata\stata\stata11_se\h_elsa.dta", clear

merge 1:1 idauniq using elsaw6biomarkers, nogen

merge 1:1 idauniq using elsaw6gor, nogen

merge 1:1 idauniq using elsaw6ifsedu, nogen

merge 1:1 idauniq using elsaw6income, nogen

keep if innursewave==1

gen cons=1

recode edqual -9/-2=. 1=1 2/7=0, gen(degree)

label define degree 0 "No degree" 1 "Degree"

label values degree degree

recode edqual -9/-2=. 1/3=1 4/7=0, gen(highlowedu)

label define highlowedu 0 "Low edu" 1 "High edu"

label values highlowedu highlowedu

gen eqtotinc_bu_s52=eqtotinc_bu_s*52

xtile eqtotinc_3cat =eqtotinc_bu_s52, nq(3)

label define eqtotinc_3cat 1 "Low income" 2 "Medium income" 3 "High income"

label values eqtotinc_3cat eqtotinc_3cat

drop if eqtotinc_3cat==.

drop if highlowedu==.

recode raracem 1=1 4=0 else=., gen(white)

label define white 1 "White" 0 "BME"

label values white white

*biomarkers

recode hba1c -8/-1=.

recode pulse1 -1 999=.

recode pulse2 -1 999=.

recode pulse3 -1 999=.

egen pulse = rowmean(pulse1 pulse2 pulse3)

replace pulse =. if pulse1==.

replace pulse =. if pulse2==.

replace pulse =. if pulse3==.

replace pulse =. if consubno==0

recode chol -6/-1=.

rename SYSVAL sysval

recode sysval -1=.

rename BMIVAL bmival

recode bmival -1=.

rename WSTVAL wstval

recode wstval -1=.

recode wstval 999.9=.

recode hscrp -6/-1=.

*missing biomarker data reasons

recode hscrp 10/1000 =.

tab refusedblood

tab clotb

tab samdifno

tab consubno

*aux variables for inputation:

recode r6mstat -18 -4=. 3 4 5 7 8=0, gen(married)

encode GOR, gen(GOR_n)

recode GOR_n 10 11=.

recode h6ahown -13 -4=., gen(ownhome)

recode Heill -9/-8=. 2=0, gen(longstandingillness)

recode Helim -8=. -1 2=0, gen(limitingillness)

recode r6shlt -18/-4=., gen(srh)

rename h6hhres hhsize

keep idauniq cons r6agey ragender white degree highlowedu eqtotinc_3cat hba1c hscrp pulse chol sysval bmival wstval consubno w6nurwt w6bldwt refusedblood clotb WSTOKB samdifno difbpcno respwh BSOUTC married GOR_n hhsize ownhome longstandingillness limitingillness srh idahhw6

save "${mystart}\.do files for NSR\mapbio_elsa_data.dta", replace

**UKHLS data preparation**

cd "${mystart}\.do files for NSR"

use "${mystart}\.do files for NSR\UKHLS\UKDA-7251-stata\stata\stata11\xindresp_ns.dta", clear

gen refusedblood=0

foreach var of varlist refbsc1 refbsc2 refbsc3 refbsc4 refbsc5 refbsc6 refbsc7 refbsc95 {

replace refusedblood=1 if `var'==1

}

keep pidp b_hidp c_hidp hhsize nsex age hiqual_dv ethni bmival wstval omsysval ompulval consubx5 indnsub_xw nurdayy bpconst difbpc1 clotb bswill samdifc1 whintro wjrel ieqmoecd_dv bsoute bmiok marstat health sf1 psu strata refusedblood

merge 1:1 pidp using "${mystart}\.do files for NSR\UKHLS\UKDA-7251-stata\stata\stata11\xlabblood_ns.dta", nogen

drop if age <50

drop if age==.

gen innurse=1

save mapbio_us_data, replace

use "${mystart}\.do files for NSR\UKHLS\UKDA-6614-stata\stata\stata11_se\ukhls_w3\c_hhresp.dta", clear

keep c_hidp c_fihhmngrs_dv c_country c_gor_dv c_tenure_dv

clonevar bc_hidp=c_hidp

save c_householdincome, replace

use "${mystart}\.do files for NSR\UKHLS\UKDA-6614-stata\stata\stata11_se\ukhls_w2\b_hhresp.dta", clear

keep b_hidp b_fihhmngrs_dv b_country b_gor_dv b_tenure_dv

clonevar bc_hidp=b_hidp

save b_householdincome, replace

merge 1:1 bc_hidp using c_householdincome

save bc_householdincome, replace

use "${mystart}\.do files for NSR\mapbio_us_data.dta"

merge m:1 b_hidp using b_householdincome, nogen

merge m:1 c_hidp using c_householdincome, nogen

clonevar bc_fihhmngrs_dv=b_fihhmngrs_dv

replace bc_fihhmngrs_dv=c_fihhmngrs_dv if bc_fihhmngrs_dv==.

clonevar bc_gor_dv=b_gor_dv

replace bc_gor_dv=c_gor_dv if bc_gor_dv==.

clonevar bc_tenure_dv=b_tenure_dv

replace bc_tenure_dv=c_tenure_dv if bc_tenure_dv==.

keep if innurse==1

*country

drop if b_country==2

drop if b_country==3

drop if c_country==2

drop if c_country==3

drop if b_country==. & c_country==.

*missing sociodems

*variables

gen cons=1

recode age 91/102=90

xtile bc_fihhmngrs_dv_3cat =bc_fihhmngrs_dv, nq(3)

gen bc_fihhmngrs_dv_equiv=bc_fihhmngrs_dv/ieqmoecd_dv

gen bc_fihhmngrs_dv_equiv_12=bc_fihhmngrs_dv_equiv*12

xtile income3 =bc_fihhmngrs_dv_equiv_12, nq(3)

label define income3 1 "Low income" 2 "Medium income" 3 "High income"

label values income3 income3

recode hiqual_dv 1=1 2/9=0 -9=., gen(degree)

label define degree 1 "Degree" 0 "No degree"

label values degree degree

recode hiqual_dv 1/3=1 4/9=0 -9=., gen(highlowedu)

label define highlowedu 1 "High edu" 0 "Low edu"

label values highlowedu highlowedu

recode ethnic 1 2 4 =1 5/97=0 else=., gen(white)

label define white 1 "White" 0 "BME"

label values white white

recode hscrp -41/-9=.

recode hba1c -42/-9=.

recode bmival -8=.

recode wstval -8=.

recode chol -41/-9=.

recode hdl -41/-9=.

recode omsysval -8=.

recode ompulval -8=.

drop if nsex==.

drop if bc_fihhmngrs_dv==.

drop if highlowedu==.

drop if white==.

*missing biomarker data reasons

recode hscrp 10/1000 =.

tab refusedblood

tab bswill

tab consubx5

*aux variables for imputation:

recode marstat -2=. 2=1 1 3 4 5 6 7 9=0, gen(married)

recode bc_gor_dv -9=.

recode bc_tenure_dv -9=. 1 2=1 3/8=0, gen(ownhome)

recode health -2/-1=. 2=0, gen(longstandingillness)

recode sf1 -1=., gen(srh)

keep pidp cons age nsex white degree highlowedu income3 hba1c chol omsysval ompulval consubx5 bmival wstval hscrp indbdub_xw indnsub_xw dory nurdayy bpconst difbpc1 clotb bswill samdifc1 whintro wjrel bsoute married bc_gor_dv hhsize ownhome longstandingillness srh bc_hidp psu strata refusedblood

save mapbio_us_data, replace

**Pooled data preparation**

use "${mystart}\.do files for NSR\mapbio_us_data.dta", clear

rename pidp id

rename nsex sex

rename omsysval sysval

rename ompulval pulse

gen survey =2

label define survey 1 "ELSA" 2 "US"

label values survey survey

gen cholminmean=chol-5.556279

gen cholstd = cholminmean/1.226908

keep id survey sex age white degree highlowedu income3 hba1c chol cholstd sysval pulse wstval bmival indbdub_xw indnsub_xw hscrp married bc_gor_dv hhsize ownhome longstandingillness srh bc_hidp psu strata

save mapbio_pooled_us, replace

use "${mystart}\.do files for NSR\mapbio_elsa_data.dta", clear

rename idauniq id

rename ragender sex

rename r6agey age

rename eqtotinc_3cat income3

gen survey =1

label define survey 1 "ELSA" 2 "US"

label values survey survey

gen cholminmean = chol -5.558676

gen cholstd = cholminmean/1.166452

keep id survey sex age white degree highlowedu income3 hba1c chol cholstd sysval pulse wstval bmival w6nurwt w6bldwt hscrp married GOR_n hhsize ownhome longstandingillness limitingillness srh idahhw6

save mapbio_pooled_elsa, replace

append using mapbio_pooled_us

gen cons=1

label drop ragender

label define sex 1 "Men" 2 "Women"

label values sex sex

recast float id, force

recast float chol, force

recast float sysval, force

recast float pulse, force

recast float wstval, force

recast float hscrp, force

recode age 50/59=1 60/69=2 70/79=3 80/100=4, gen(age_10)

gen age2 = age*age

egen intersections1 = group(sex white highlowedu income3)

egen justone1 = tag(intersections1)

egen intersections2 = group(sex white highlowedu income3 age_10)

egen justone2 = tag(intersections1)

egen hba1c_std = std(hba1c)

egen chol_std = std(chol)

egen sysval_std = std(sysval)

egen pulse_std = std(pulse)

egen wstval_std = std(wstval)

egen bmival_std = std(bmival)

egen hscrp_std = std(hscrp)

gen bloodweight=.

gen nurseweight=.

replace bloodweight=w6bldwt

replace bloodweight= indbdub_xw if bloodweight==.

replace nurseweight= w6nurwt

replace nurseweight= indnsub_xw if nurseweight==.

save mapbio_pooled_data, replace

**Main analysis**

cd "${mystart}\.do files for NSR"

use mapbio_pooled_data, clear

sort intersections1 id

*Table 1 Sample characteristics

sum age if survey==1

sum age if survey==2

sum age

foreach var of varlist sex white highlowedu income3 {

tab `var' if survey==1

tab `var' if survey==2

tab `var'

}

foreach var of varlist hba1c chol hscrp sysval pulse bmival {

sum `var' if survey==1

sum `var' if survey==2

sum `var'

}

foreach var of varlist hba1c chol hscrp sysval pulse bmival {

tab `var' if survey==1, m

tab `var' if survey==2, m

tab `var', m

}

*Table 2 Coefficient estimates from linear regression main effects models

reg hba1c i.sex b1.white b1.highlowedu b3.income3 c.age c.age2, cformat(%3.2f)

reg chol i.sex b1.white b1.highlowedu b3.income3 c.age c.age2, cformat(%3.2f)

reg hscrp i.sex b1.white b1.highlowedu b3.income3 c.age c.age2, cformat(%3.2f)

reg sysval i.sex b1.white b1.highlowedu b3.income3 c.age c.age2, cformat(%3.2f)

reg pulse i.sex b1.white b1.highlowedu b3.income3 c.age c.age2, cformat(%3.2f)

reg bmival i.sex b1.white b1.highlowedu b3.income3 c.age c.age2, cformat(%3.2f)

*To obtain results for age2

reg hba1c i.sex b1.white b1.highlowedu b3.income3 c.age c.age2

reg chol i.sex b1.white b1.highlowedu b3.income3 c.age c.age2

reg hscrp i.sex b1.white b1.highlowedu b3.income3 c.age c.age2

reg sysval i.sex b1.white b1.highlowedu b3.income3 c.age c.age2

reg pulse i.sex b1.white b1.highlowedu b3.income3 c.age c.age2

reg bmival i.sex b1.white b1.highlowedu b3.income3 c.age c.age2

*Table 3 Disparities in biomarkers across intersections

*Constructed manually using predicted marginal effects

quietly reg hba1c i.sex b1.white b1.highlowedu b3.income3 c.age c.age2

margins i.sex#i.white#i.highlowedu#i.income3

quietly reg chol i.sex b1.white b1.highlowedu b3.income3 c.age c.age2

margins i.sex#i.white#i.highlowedu#i.income3

quietly reg hscrp i.sex b1.white b1.highlowedu b3.income3 c.age c.age2

margins i.sex#i.white#i.highlowedu#i.income3

quietly reg sysval i.sex b1.white b1.highlowedu b3.income3 c.age c.age2

margins i.sex#i.white#i.highlowedu#i.income3

quietly reg pulse i.sex b1.white b1.highlowedu b3.income3 c.age c.age2

margins i.sex#i.white#i.highlowedu#i.income3

quietly reg bmival i.sex b1.white b1.highlowedu b3.income3 c.age c.age2

margins i.sex#i.white#i.highlowedu#i.income3

*Figure 1 Flow chart diagram

*Constructed manually using information on dropped cases for data .do files

*Figure 2

*Constructed manually (no empirical data)

*Figures 3 and 4

*Constructed manually by exporting predicted marginal effects and SEs into intersectionsgraphs.dta and running .do file intersectional graphs_nsr.do

Supplementary table 1 Sample characteristics (weighted data)

*ELSA

svyset idahhw6 [pweight=w6nurwt], strata(GOR_n)

svy: mean age if survey==1

estat sd

foreach var of varlist sex white highlowedu income3 {

svy: tab `var' if survey==1

svy: tab `var' if survey==1, count

}

foreach var of varlist sysval pulse bmival {

quietly svy: mean `var' if survey==1

estat sd

}

foreach var of varlist sysval pulse bmival {

svy: tab `var' if survey==1, count

}

svyset idahhw6 [pweight=w6bldwt], strata(GOR_n)

foreach var of varlist hba1c chol hscrp {

quietly svy: mean `var' if survey==1

estat sd

}

foreach var of varlist hba1c chol hscrp {

svy: tab `var' if survey==1, count

}

*UKHLS

svyset psu [pweight=indnsub_xw], strata(strata) single(scaled)

svy: mean age if survey==2

estat sd

foreach var of varlist sex white highlowedu income3 {

svy: tab `var' if survey==2

svy: tab `var' if survey==2, count

}

foreach var of varlist sysval pulse bmival {

quietly svy: mean `var' if survey==2

estat sd

}

foreach var of varlist sysval pulse bmival {

svy: tab `var' if survey==2, count

}

svyset psu [pweight=indbdub_xw], strata(strata) single(scaled)

foreach var of varlist hba1c chol hscrp {

quietly svy: mean `var' if survey==2

estat sd

}

foreach var of varlist hba1c chol hscrp {

svy: tab `var' if survey==2, count

}

*Supplementary table 2 Summary of multilevel and linear regression models

*Multilevel models, null

sort intersections1

foreach var of varlist hba1c_std chol_std hscrp_std sysval_std pulse_std bmival_std {

quietly runmlwin `var' c.age c.age2 cons, level2(intersections1: cons) level1(id: cons) nopause

estat ic

display [RP2]var(cons)/([RP2]var(cons) + [RP1]var(cons)) //estimate icc/vpc

}

*Multilevel models, main effects

foreach var of varlist hba1c_std chol_std hscrp_std sysval_std pulse_std bmival_std {

quietly runmlwin `var' i.sex i.white i.highlowedu i.income3 c.age c.age2 cons, level2(intersections1: cons) level1(id: cons) nopause

estat ic

display [RP2]var(cons)/([RP2]var(cons) + [RP1]var(cons)) //estimate icc/vpc

}

*Standardised BIC values linear regression

quietly reg hba1c_std i.sex b1.white b1.highlowedu b3.income3 c.age c.age2, cformat(%3.2f)

estat ic

quietly reg chol_std i.sex b1.white b1.highlowedu b3.income3 c.age c.age2, cformat(%3.2f)

estat ic

quietly reg hscrp_std i.sex b1.white b1.highlowedu b3.income3 c.age c.age2, cformat(%3.2f)

estat ic

quietly reg sysval_std i.sex b1.white b1.highlowedu b3.income3 c.age c.age2, cformat(%3.2f)

estat ic

quietly reg pulse_std i.sex b1.white b1.highlowedu b3.income3 c.age c.age2, cformat(%3.2f)

estat ic

quietly reg bmival_std i.sex b1.white b1.highlowedu b3.income3 c.age c.age2, cformat(%3.2f)

estat ic

*Supplementary table 3 Summary of multilevel models including age-defined intersections

*Multilevel models, null

sort intersections2 id

foreach var of varlist hba1c_std chol_std hscrp_std sysval_std pulse_std bmival_std {

runmlwin `var' cons, level2(intersections2: cons) level1(id: cons) nopause

estat ic

display [RP2]var(cons)/([RP2]var(cons) + [RP1]var(cons)) //estimate icc/vpc

runmlwin `var' cons, level2(intersections2: cons) level1(id: cons) nopause mcmc(burnin(5000) chain(50000)) initsprevious

display [RP2]var(cons)/([RP2]var(cons) + [RP1]var(cons)) //estimate icc/vpc

}

*Multilevel models, main effects

sort intersections2 id

foreach var of varlist hba1c_std chol_std hscrp_std sysval_std pulse_std bmival_std {

runmlwin `var' i.sex i.white i.highlowedu i.income3 i.age_10 cons, level2(intersections2: cons) level1(id: cons) nopause

estat ic

display [RP2]var(cons)/([RP2]var(cons) + [RP1]var(cons)) //estimate icc/vpc

runmlwin `var' i.sex i.white i.highlowedu i.income3 i.age_10 cons, level2(intersections2: cons) level1(id: cons) nopause mcmc(burnin(5000) chain(50000)) initsprevious

display [RP2]var(cons)/([RP2]var(cons) + [RP1]var(cons)) //estimate icc/vpc

}

*Supplementary table 4 Intersectional subgroups used in analysis

tab intersections1

tab intersections1 if survey==1

tab intersections1 if survey==2

*Supplementary table 5 Coefficient estimates from linear regression main effects models with standardised outcomes

reg hba1c_std i.sex b1.white b1.highlowedu b3.income3 c.age c.age2, cformat(%3.2f)

reg chol_std i.sex b1.white b1.highlowedu b3.income3 c.age c.age2, cformat(%3.2f)

reg hscrp_std i.sex b1.white b1.highlowedu b3.income3 c.age c.age2, cformat(%3.2f)

reg sysval_std i.sex b1.white b1.highlowedu b3.income3 c.age c.age2, cformat(%3.2f)

reg pulse_std i.sex b1.white b1.highlowedu b3.income3 c.age c.age2, cformat(%3.2f)

reg bmival_std i.sex b1.white b1.highlowedu b3.income3 c.age c.age2, cformat(%3.2f)

*To obtain results for age2

reg hba1c_std i.sex b1.white b1.highlowedu b3.income3 c.age c.age2

reg chol_std i.sex b1.white b1.highlowedu b3.income3 c.age c.age2

reg hscrp_std i.sex b1.white b1.highlowedu b3.income3 c.age c.age2

reg sysval_std i.sex b1.white b1.highlowedu b3.income3 c.age c.age2

reg pulse_std i.sex b1.white b1.highlowedu b3.income3 c.age c.age2

reg bmival_std i.sex b1.white b1.highlowedu b3.income3 c.age c.age2

*Supplementary figure 1 Gender, ethnic, education and income inequalities in healthy ageing biomarkers – ELSA and UKHLS

reg hba1c c.age c.age2 i.sex b1.white b1.highlowedu b3.income3 if survey==1

estimates store hba1c1

reg hba1c c.age c.age2 i.sex b1.white b1.highlowedu b3.income3 if survey==2

estimates store hba1c2

reg hba1c c.age c.age2 i.sex b1.white b1.highlowedu b3.income3

estimates store hba1c3

reg chol c.age c.age2 i.sex b1.white b1.highlowedu b3.income3 if survey==1

estimates store chol1

reg chol c.age c.age2 i.sex b1.white b1.highlowedu b3.income3 if survey==2

estimates store chol2

reg chol c.age c.age2 i.sex b1.white b1.highlowedu b3.income3

estimates store chol3

reg hscrp c.age c.age2 i.sex b1.white b1.highlowedu b3.income3 if survey==1

estimates store hscrp1

reg hscrp c.age c.age2 i.sex b1.white b1.highlowedu b3.income3 if survey==2

estimates store hscrp2

reg hscrp c.age c.age2 i.sex b1.white b1.highlowedu b3.income3

estimates store hscrp3

reg sysval c.age c.age2 i.sex b1.white b1.highlowedu b3.income3 if survey==1

estimates store sysval1

reg sysval c.age c.age2 i.sex b1.white b1.highlowedu b3.income3 if survey==2

estimates store sysval2

reg sysval c.age c.age2 i.sex b1.white b1.highlowedu b3.income3

estimates store sysval3

reg pulse c.age c.age2 i.sex b1.white b1.highlowedu b3.income3 if survey==1

estimates store pulse1

reg pulse c.age c.age2 i.sex b1.white b1.highlowedu b3.income3 if survey==2

estimates store pulse2

reg pulse c.age c.age2 i.sex b1.white b1.highlowedu b3.income3

estimates store pulse3

reg bmival c.age c.age2 i.sex b1.white b1.highlowedu b3.income3 if survey==1

estimates store bmival1

reg bmival c.age c.age2 i.sex b1.white b1.highlowedu b3.income3 if survey==2

estimates store bmival2

reg bmival c.age c.age2 i.sex b1.white b1.highlowedu b3.income3

estimates store bmival3

coefplot (hba1c1, label(ELSA)) (hba1c2, label(UKHLS)) (hba1c3, label(Pooled)), bylabel(HbA1c (mmol/mol)) || (chol1) (chol2) (chol3), bylabel(Cholesterol (mmol/L)) ///

|| (hscrp1) (hscrp2) (hscrp3), bylabel(CRP (mg/L)) || (sysval1) (sysval2) (sysval3), bylabel(SBP (mm Hg)) ///

|| (pulse1) (pulse2) (pulse3), bylabel(RHR (bpm)) || (bmival1) (bmival2) (bmival3), bylabel(BMI (kg/m2)) xline(0) drop(_cons age age2) ///

byopts(xrescale) order(sex white highlowedu income3) msymbol(square) msize(small) scheme(plottig)

graph export surveycompare.tif, replace

*Supplementary Figure 2. see missing multiple imputation_nsr

*Supplementary Figure 3. see weights_nsr

**Intersectional graphs**

cd "${mystart}\.do files for NSR"

use "${mystart}\.do files for NSR\intersectionsgraphs.dta", clear

*hba1c

serrbar hba1c_margin hba1c_se hba1c_rank , scale(1.96)yline(40.32054) ylabel(, ang(90)) ytitle("HbA1c (mmol/mol)") scheme(plottig) xlabel( ///

1 "White women high education high income" ///

2 "White men high education high income" ///

3 "White women low education high income" ///

4 "White women high education medium income" ///

5 "White men low education high income" ///

6 "White women high education low income" ///

7 "White men high education medium income" ///

8 "White men high education low income" ///

9 "White women low education medium income" ///

10 "White women low education low income" ///

11 "White men low education medium income" ///

12 "White men low education low income" ///

13 "BME women high education high income" ///

14 "BME men high education high income" ///

15 "BME women low education high income" ///

16 "BME women high education medium income" ///

17 "BME men low education high income" ///

18 "BME women high education low income" ///

19 "BME men high education medium income" ///

20 "BME men high education low income" ///

21 "BME women low education medium income" ///

22 "BME women low education low income" ///

23 "BME men low education medium income" ///

24 "BME men low education low income" ///

, angle(90) labsize(vsmall)) xtitle("")

graph export hba1c.tif, replace

*chol

serrbar chol_margin chol_se chol_rank, scale(1.96)yline(5.534294) ylabel(, ang(90)) ytitle("Cholesterol (mmol/L)") scheme(plottig) xlabel( ///

1 "BME men low education low income" ///

2 "BME men low education medium income" ///

3 "BME men high education low income" ///

4 "BME men high education medium income" ///

5 "BME men low education high income" ///

6 "BME men high education high income" ///

7 "White men low education low income" ///

8 "White men low education medium income" ///

9 "White men high education low income" ///

10 "White men high education medium income" ///

11 "White men low education high income" ///

12 "BME women low education low income" ///

13 "BME women low education medium income" ///

14 "White men high education high income" ///

15 "BME women high education low income" ///

16 "BME women high education medium income" ///

17 "BME women low education high income" ///

18 "BME women high education high income" ///

19 "White women low education low income" ///

20 "White women low education medium income" ///

21 "White women high education low income" ///

22 "White women high education medium income" ///

23 "White women low education high income" ///

24 "White women high education high income" ///

, angle(90) labsize(vsmall)) xtitle("")

graph export chol.tif, replace

*crp

serrbar crp_margin crp_se crp_rank, scale(1.96)yline(2.178585) ylabel(, ang(90)) ytitle("CRP (mg/L)") scheme(plottig) xlabel( ///

1 "BME men high education high income" ///

2 "White men high education high income" ///

3 "BME men high education medium income" ///

4 "BME women high education high income" ///

5 "BME men low education high income" ///

6 "White men high education medium income" ///

7 "White women high education high income" ///

8 "White men low education high income" ///

9 "BME men high education low income" ///

10 "White men high education low income" ///

11 "BME women high education medium income" ///

12 "BME men low education medium income" ///

13 "BME women low education high income" ///

14 "White women high education medium income" ///

15 "White men low education medium income" ///

16 "White women low education high income" ///

17 "BME women high education low income" ///

18 "BME men low education low income" ///

19 "White women high education low income" ///

20 "White men low education low income" ///

21 "BME women low education medium income" ///

22 "White women low education medium income" ///

23 "BME women low education low income" ///

24 "White women low education low income" ///

, angle(90) labsize(vsmall)) xtitle("")

graph export crp.tif, replace

*sysval

serrbar sysval_margin sysval_se sysval_rank, scale(1.96)yline(131.8054) ylabel(, ang(90)) ytitle("SBP (mm Hg)") scheme(plottig) xlabel( ///

1 "White women high education medium income" ///

2 "White women high education high income" ///

3 "White women high education low income" ///

4 "White women low education medium income" ///

5 "BME women high education medium income" ///

6 "White women low education high income" ///

7 "White women low education low income" ///

8 "BME women high education high income" ///

9 "BME women high education low income" ///

10 "White men high education medium income" ///

11 "BME women low education medium income" ///

12 "White men high education high income" ///

13 "White men high education low income" ///

14 "BME women low education high income" ///

15 "BME women low education low income" ///

16 "White men low education medium income" ///

17 "BME men high education medium income" ///

18 "White men low education high income" ///

19 "White men low education low income" ///

20 "BME men high education high income" ///

21 "BME men high education low income" ///

22 "BME men low education medium income" ///

23 "BME men low education high income" ///

24 "BME men low education low income" ///

, angle(90) labsize(vsmall)) xtitle("")

graph export sysval.tif, replace

*pulse

serrbar pulse_margin pulse_se pulse_rank, scale(1.96)yline(67.50258) ylabel(, ang(90)) ytitle("RHR (bpm)") scheme(plottig) xlabel( ///

1 "White men high education high income" ///

2 "White men low education high income" ///

3 "White men high education medium income" ///

4 "White men high education low income" ///

5 "BME men high education high income" ///

6 "White men low education medium income" ///

7 "White men low education low income" ///

8 "BME men low education high income" ///

9 "BME men high education medium income" ///

10 "White women high education high income" ///

11 "BME men high education low income" ///

12 "BME men low education medium income" ///

13 "White women low education high income" ///

14 "White women high education medium income" ///

15 "BME men low education low income" ///

16 "White women high education low income" ///

17 "BME women high education high income" ///

18 "White women low education medium income" ///

19 "White women low education low income" ///

20 "BME women low education high income" ///

21 "BME women high education medium income" ///

22 "BME women high education low income" ///

23 "BME women low education medium income" ///

24 "BME women low education low income" ///

, angle(90) labsize(vsmall)) xtitle("")

graph export pulse.tif, replace

*bmi

serrbar bmival_margin bmival_se bmival_rank, scale(1.96)yline(28.42972) ylabel(, ang(90)) ytitle("BMI (kg/m2)") scheme(plottig) xlabel( ///

1 "White women high education high income" ///

2 "White men high education high income" ///

3 "BME women high education high income" ///

4 "BME men high education high income" ///

5 "White women high education medium income" ///

6 "White women high education low income" ///

7 "White men high education medium income" ///

8 "White men high education low income" ///

9 "White women low education high income" ///

10 "BME women high education medium income" ///

11 "White men low education high income" ///

12 "BME women high education low income" ///

13 "BME men high education medium income" ///

14 "BME men high education low income" ///

15 "BME women low education high income" ///

16 "BME men low education high income" ///

17 "White women low education medium income" ///

18 "White women low education low income" ///

19 "White men low education medium income" ///

20 "White men low education low income" ///

21 "BME women low education medium income" ///

22 "BME women low education low income" ///

23 "BME men low education medium income" ///

24 "BME men low education low income" ///

, angle(90) labsize(vsmall)) xtitle("")

graph export bmi.tif, replace

**Multiple imputation**

*for ELSA

cd "${mystart}\.do files for NSR"

use mapbio_pooled_data, clear

keep if survey==1

drop if sex==.

drop if white==.

drop if highlowedu==.

drop if income3==.

drop if age==.

drop if married==.

drop if GOR_n==.

drop if hhsize==.

drop if ownhome==.

drop if limitingillness==.

drop if srh==.

mi set wide

mi xtset, clear

mi register imputed hba1c chol hscrp sysval pulse bmival

mi register passive sex white highlowedu income3 age age2

mi impute chained (reg) hba1c chol hscrp sysval bmival pulse = sex white highlowedu income3 age age2 married GOR_n hhsize ownhome limitingillness srh, add(25) dots rseed (1234) burn(100) noisily

save mapbio_elsa_data_imputed, replace

*for UKHLS

cd "${mystart}\.do files for NSR"

use mapbio_pooled_data, clear

keep if survey==2

drop if sex==.

drop if white==.

drop if highlowedu==.

drop if income3==.

drop if age==.

drop if married==.

drop if bc_gor_dv==.

drop if hhsize==.

drop if ownhome==.

drop if longstandingillness==.

drop if srh==.

mi set wide

mi xtset, clear

mi register imputed hba1c chol hscrp sysval pulse bmival

mi register passive sex white highlowedu income3 age age2 married bc_gor_dv hhsize ownhome longstandingillness srh

mi impute chained (reg) hba1c chol hscrp sysval bmival pulse = sex white highlowedu income3 age age2 married bc_gor_dv hhsize ownhome longstandingillness srh, add(39) dots rseed (1234) burn(100) noisily

save mapbio_us_data_imputed, replace

*for pooled data

cd "${mystart}\.do files for NSR"

use mapbio_pooled_data, clear

drop if sex==.

drop if white==.

drop if highlowedu==.

drop if income3==.

drop if age==.

drop if married==.

drop if hhsize==.

drop if ownhome==.

drop if longstandingillness==.

drop if srh==.

mi set wide

mi xtset, clear

mi register imputed hba1c chol hscrp sysval pulse bmival

mi register passive sex white highlowedu income3 age age2 married hhsize ownhome longstandingillness srh survey

mi impute chained (reg) hba1c chol hscrp sysval bmival pulse = sex white highlowedu income3 age age2 married hhsize ownhome longstandingillness srh survey, add(34) dots rseed (1234) burn(100) noisily

save mapbio_pooled_data_imputed, replace

*Graph

reg hba1c i.sex b1.white b1.highlowedu b3.income3 c.age c.age2, cformat(%3.2f)

estimates store hba1c1

mi est, cformat(%3.2f): reg hba1c i.sex b1.white b1.highlowedu b3.income3 c.age c.age2

estimates store hba1c2

reg chol i.sex b1.white b1.highlowedu b3.income3 c.age c.age2, cformat(%3.2f)

estimates store chol1

mi est, cformat(%3.2f): reg chol i.sex b1.white b1.highlowedu b3.income3 c.age c.age2

estimates store chol2

reg hscrp i.sex b1.white b1.highlowedu b3.income3 c.age c.age2, cformat(%3.2f)

estimates store crp1

mi est, cformat(%3.2f): reg hscrp i.sex b1.white b1.highlowedu b3.income3 c.age c.age2

estimates store crp2

reg sysval i.sex b1.white b1.highlowedu b3.income3 c.age c.age2, cformat(%3.2f)

estimates store sysval1

mi est, cformat(%3.2f): reg sysval i.sex b1.white b1.highlowedu b3.income3 c.age c.age2

estimates store sysval2

reg pulse i.sex b1.white b1.highlowedu b3.income3 c.age c.age2, cformat(%3.2f)

estimates store pulse1

mi est, cformat(%3.2f): reg pulse i.sex b1.white b1.highlowedu b3.income3 c.age c.age2

estimates store pulse2

reg bmival i.sex b1.white b1.highlowedu b3.income3 c.age c.age2, cformat(%3.2f)

estimates store bmival1

mi est, cformat(%3.2f): reg bmival i.sex b1.white b1.highlowedu b3.income3 c.age c.age2

estimates store bmival2

coefplot (hba1c1, label(Non-imputed data)) (hba1c2, label(Imputed data)), bylabel(HbA1c (mmol/mol)) || (chol1, label(Non-imputed data)) (chol2, label(Imputed data)), bylabel(Cholesterol (mmol/L)) ///

|| (crp1, label(Non-imputed data)) (crp2, label(Imputed data)), bylabel(CRP (mg/L)) || (sysval1, label(Non-imputed data)) (sysval2, label(Imputed data)), bylabel(SBP (mm Hg)) ///

|| (pulse1, label(Non-imputed data)) (pulse2, label(Imputed data)), bylabel(RHR (bpm)) || (bmival1, label(Non-imputed data)) (bmival2, label(Imputed data)), bylabel(BMI (kg/m2)) xline(0) drop(_cons age age2) ///

byopts(xrescale) order(sex white highlowedu income3) msymbol(square) msize(small) scheme(plottig)

graph export imputationcompare.tif, replace

**Weights**

cd "${mystart}\.do files for NSR"

use mapbio_pooled_data, clear

*ELSA

svyset idahhw6 [pweight=w6bldwt], strata(GOR_n)

reg hba1c i.sex b1.white b1.highlowedu b3.income3 c.age c.age2 if survey==1

estimates store hba1c1

svy: reg hba1c i.sex b1.white b1.highlowedu b3.income3 c.age c.age2

estimates store hba1c2

reg chol i.sex b1.white b1.highlowedu b3.income3 c.age c.age2 if survey==1

estimates store chol1

svy: reg chol i.sex b1.white b1.highlowedu b3.income3 c.age c.age2

estimates store chol2

reg hscrp i.sex b1.white b1.highlowedu b3.income3 c.age c.age2 if survey==1

estimates store hscrp1

svy: reg hscrp i.sex b1.white b1.highlowedu b3.income3 c.age c.age2

estimates store hscrp2

svyset idahhw6 [pweight=w6nurwt], strata(GOR_n)

reg sysval i.sex b1.white b1.highlowedu b3.income3 c.age c.age2 if survey==1

estimates store sysval1

svy: reg sysval i.sex b1.white b1.highlowedu b3.income3 c.age c.age2

estimates store sysval2

reg pulse i.sex b1.white b1.highlowedu b3.income3 c.age c.age2 if survey==1

estimates store pulse1

svy: reg pulse i.sex b1.white b1.highlowedu b3.income3 c.age c.age2

estimates store pulse2

reg bmival i.sex b1.white b1.highlowedu b3.income3 c.age c.age2 if survey==1

estimates store bmival1

svy: reg bmival i.sex b1.white b1.highlowedu b3.income3 c.age c.age2

estimates store bmival2

*UKHLS

svyset psu [pweight=indbdub_xw], strata(strata) single(scaled)

reg hba1c i.sex b1.white b1.highlowedu b3.income3 c.age c.age2 if survey==2

estimates store hba1c3

svy: reg hba1c i.sex b1.white b1.highlowedu b3.income3 c.age c.age2

estimates store hba1c4

reg chol i.sex b1.white b1.highlowedu b3.income3 c.age c.age2 if survey==2

estimates store chol3

svy: reg chol i.sex b1.white b1.highlowedu b3.income3 c.age c.age2

estimates store chol4

reg hscrp i.sex b1.white b1.highlowedu b3.income3 c.age c.age2 if survey==2

estimates store hscrp3

svy: reg hscrp i.sex b1.white b1.highlowedu b3.income3 c.age c.age2

estimates store hscrp4

svyset psu [pweight=indnsub_xw], strata(strata) single(scaled)

reg sysval i.sex b1.white b1.highlowedu b3.income3 c.age c.age2 if survey==2

estimates store sysval3

svy: reg sysval i.sex b1.white b1.highlowedu b3.income3 c.age c.age2

estimates store sysval4

reg pulse i.sex b1.white b1.highlowedu b3.income3 c.age c.age2 if survey==2

estimates store pulse3

svy: reg pulse i.sex b1.white b1.highlowedu b3.income3 c.age c.age2

estimates store pulse4

reg bmival i.sex b1.white b1.highlowedu b3.income3 c.age c.age2 if survey==2

estimates store bmival3

svy: reg bmival i.sex b1.white b1.highlowedu b3.income3 c.age c.age2

estimates store bmival4

coefplot (hba1c1, label(ELSA unweighted)) (hba1c2, label(ELSA weighted)) (hba1c3, label(UKHLS unweighted)) (hba1c4, label(UKHLS weighted)), bylabel(HbA1c (mmol/mol)) || (chol1, label(ELSA unweighted)) (chol2, label(ELSA weighted)) (chol3, label(UKHLS unweighted)) (chol4, label(UKHLS weighted)), bylabel(Cholesterol (mmol/L)) ///

|| (hscrp1, label(ELSA unweighted)) (hscrp2, label(ELSA weighted)) (hscrp3, label(UKHLS unweighted)) (hscrp4, label(UKHLS weighted)), bylabel(CRP (mg/L)) || (sysval1, label(ELSA unweighted)) (sysval2, label(ELSA weighted)) (sysval3, label(UKHLS unweighted)) (sysval4, label(UKHLS weighted)), bylabel(SBP (mm Hg)) ///

|| (pulse1, label(ELSA unweighted)) (pulse2, label(ELSA weighted)) (pulse3, label(UKHLS unweighted)) (pulse4, label(UKHLS weighted)), bylabel(RHR (bpm)) || (bmival1, label(ELSA unweighted)) (bmival2, label(ELSA weighted)) (bmival3, label(UKHLS unweighted)) (bmival4, label(UKHLS weighted)), bylabel(BMI (kg/m2)) xline(0) drop(_cons age age2) ///

byopts(xrescale) order(sex white highlowedu income3) msymbol(square) msize(small) scheme(plottig)

graph export weightscompare.tif
